# Supplementary material for: Artificial intelligence-supported diabetic retinopathy screening in Tanzania: rationale and design of a randomised controlled trial
Source: BMJ Open. 2024 Jan 25;14(1):e075055. doi: 10.1136/bmjopen-2023-075055 (PMC10824006; doi:10.1136/bmjopen-2023-075055)
Supplement: Supplementary data [file bmjopen-2023-075055supp001.pdf]

Adult Consent Form

Study Title:
Artificial Intelligence Supported Diabetic Retinopathy Screening in Tanzania

- I confirm that I have read the information sheet dated 30/5/2022 (version 1.1) for the above study. I have had the opportunity to consider the information, ask questions and have had these answered satisfactorily. ☐
- I understand that my participation is voluntary and that I am free to withdraw at any time without giving any reason, without my medical care or legal rights being affected. ☐
- I understand that the information/retinal photographs collected about me may be used to support other research in the future, and may be shared anonymously with other researchers. ☐
- I agree to receive individual feedback about my condition ☐
- I understand that the information held and maintained by the Kilimanjaro Christian Medical Centre eye department may be used to help contact me or provide information about my health status. ☐
- I agree to take part in the above study. ☐

Participant:

Signature: ……………………………………………………………………………………

Print name: ……………………………………………………………………………………..

Date:……………………………………………………………………………………………

AI-supported DR screening in Tanzania – main trial consent form – V1.1 – 30/05/2022

National Health Research Ethics Review Committee
National Institute for Medical Research
2448 Ocean Road, P.O. Box 9653
Dar es Salaam, Tanzania
Tel: +255 22 2121400
Website: www.nimr.or.tz

Witness:

Signature: ……………………………………………………………………………………

Print name: …………………………………………………………………………………..

Date:……………………………………………………………………………………………

Second witness (if required):

Signature: ……………………………………………………………………………………

Print name: …………………………………………………………………………………..

Date:……………………………………………………………………………………………

AI-supported DR screening in Tanzania – main trial consent form – V1.1 – 30/05/2022

National Health Research Ethics Review Committee
National Institute for Medical Research
2448 Ocean Road, P.O. Box 9653
Dar es Salaam, Tanzania
Tel: +255 22 2121400
Website: www.nimr.or.tz
