# Supplementary material for: Artificial intelligence-supported diabetic retinopathy screening in Tanzania: rationale and design of a randomised controlled trial
Source: BMJ Open. 2024 Jan 25;14(1):e075055. doi: 10.1136/bmjopen-2023-075055 (PMC10824006; doi:10.1136/bmjopen-2023-075055)
Supplement: Author's manuscript [file bmjopen-2023-075055.draft_revisions.pdf]

BMJ Open

BMJ Open is committed to open peer review. As part of this commitment we make the peer review history of every article we publish publicly available.

When an article is published we post the peer reviewers' comments and the authors' responses online. We also post the versions of the paper that were used during peer review. These are the versions that the peer review comments apply to.

The versions of the paper that follow are the versions that were submitted during the peer review process. They are not the versions of record or the final published versions. They should not be cited or distributed as the published version of this manuscript.

BMJ Open is an open access journal and the full, final, typeset and author-corrected version of record of the manuscript is available on our site with no access controls, subscription charges or pay-per-view fees (http://bmjopen.bmj.com).

If you have any questions on BMJ Open's open peer review process please email info.bmjopen@bmj.com

BMJ Open

Artificial Intelligence-Supported Diabetic Retinopathy Screening in Tanzania: rationale and design of a randomised controlled trial

Journal:	*BMJ Open*
Manuscript ID	bmjopen-2023-075055
Article Type:	Protocol
Date Submitted by the Author:	25-Apr-2023
Complete List of Authors:	Cleland, Charles; London School of Hygiene & Tropical Medicine, International Centre for Eye Health; Kilimanjaro Christian Medical Centre, Eye Department<br>Bascaran, Covadonga ; London School of Hygiene and Tropical Medicine International Centre for Eye Health,<br>Makupa, William; Kilimanjaro Christian Medical Centre, Eye Department<br>Shilio, Bernadetha; Union Government of Tanzania Ministry of Health Community Development Gender Elderly Children<br>Sandi, Frank; University of Dodoma School of Medicine and Nursing, Ophthalmology<br>Philippin, Heiko; London School of Hygiene & Tropical Medicine, International Centre for Eye Health; University of Freiburg Faculty of Medicine, Eye Centre<br>Marques, Ana Patricia; London School of Hygiene and Tropical Medicine International Centre for Eye Health,<br>Egan, Catherine; National Institute for Health and Care Research (NIHR) Biomedical Research Centre (BRC) for Ophthalmology, Moorfields Hospital London NHS Foundation Trust and Institute of Ophthalmology, University College London<br>Tufail, Adnan; National Institute for Health and Care Research (NIHR) Biomedical Research Centre (BRC) for Ophthalmology, Moorfields Hospital London NHS Foundation Trust and Institute of Ophthalmology, University College London<br>Keane, Pearse; National Institute for Health and Care Research (NIHR) Biomedical Research Centre (BRC) for Ophthalmology, Moorfields Hospital London NHS Foundation Trust and Institute of Ophthalmology, University College London<br>Denniston, Alastair; National Institute for Health and Care Research (NIHR) Biomedical Research Centre (BRC) for Ophthalmology, Moorfields Hospital London NHS Foundation Trust and Institute of Ophthalmology, University College London; National Institute for Health and Care Research, Birmingham Biomedical Research Centre<br>Macleod, David; London School of Hygiene & Tropical Medicine Department of Infectious Disease Epidemiology<br>Burton, Matthew J; London School of Hygiene & Tropical Medicine, International Centre for Eye Health; National Institute for Health and Care Research (NIHR) Biomedical Research Centre (BRC) for Ophthalmology, Moorfields Hospital London NHS Foundation Trust and Institute of Ophthalmology, University College London

Keywords:	Diabetic retinopathy < DIABETES & ENDOCRINOLOGY, PUBLIC HEALTH, Clinical Trial, OPHTHALMOLOGY

SCHOLARONE™ Manuscripts

Artificial Intelligence-Supported Diabetic Retinopathy Screening in Tanzania: rationale and design of a randomised controlled trial

Dr Charles R. Cleland[1,2], Dr Covadonga Bascaran[1], Dr William Makupa[2], Dr Bernadetha Shilio[3], Dr Frank Sandi[4], Dr Heiko Philippin[1,5], Dr Ana Patricia Marques[1], Dr Catherine Egan[6], Professor Adnan Tufail[6], Professor Pearse Keane[6], Professor Alastair K. Denniston[6,7,8], Dr David Macleod[9], Professor Matthew J. Burton[1,6]

[1] International Centre for Eye Health, London School of Hygiene and Tropical Medicine, London, UK

[2] Eye Department, Kilimanjaro Christian Medical Centre, Moshi, Tanzania

[3] Ministry of Health, Dodoma, Tanzania

[4] The University of Dodoma, Dodoma, Tanzania

[5] Eye Center, Medical Center, Faculty of Medicine, University of Freiburg, Germany

[6] National Institute for Health and Care Research (NIHR) Biomedical Research Centre (BRC) for Ophthalmology, Moorfields Hospital London NHS Foundation Trust and Institute of Ophthalmology, University College London, London, UK

[7] National Institute for Health and Care Research Birmingham Biomedical Research Centre, Birmingham, UK

[8] Academic Unit of Ophthalmology, Institute of Inflammation and Ageing, College of Medical and Dental Sciences, University of Birmingham, Birmingham, UK

[9] Department of Infectious Disease Epidemiology, Faculty of Epidemiology and Population Health, London School of Hygiene and Tropical Medicine, London, UK

Corresponding author: Dr Charles R Cleland

Address: International Centre for Eye Health, London School of Hygiene & Tropical Medicine, Keppel Street, London. WC1E 7HT

Email: charles.cleland@lshtm.ac.uk

Telephone number: +44 (0)20 7927 2329

Word count: 3813 words

For peer review only

ABSTRACT

Introduction

Globally diabetic retinopathy (DR) is a major cause of blindness. Sub-Saharan Africa is projected to see the largest proportionate increase in the number of people living with diabetes over the next two decades. Screening for DR is recommended to prevent sight loss; however, in many low- and middle-income countries, because of a lack of specialist eye care staff, current screening services for DR are not optimal. The use of artificial intelligence (AI) for DR screening, which automates the grading of retinal photographs and provides a point-of-screening result, offers an innovative potential solution to improve DR screening in Tanzania.

Methods and analysis

We will test the hypothesis that AI-supported DR screening increases the proportion of persons with true referable DR that attend the central ophthalmology clinic following referral after screening in a single- masked, parallel group individually randomised controlled trial. Participants will be randomised (1:1 ratio) to either AI-supported or the standard of care DR screening pathway. Participants allocated to the AI-supported screening pathway will receive their result followed by point-of-screening counselling immediately after retinal image capture. Participants in the standard of care arm will receive their result and counselling by phone once the retinal images have been graded in the usual way (typically after 2-3 weeks). The primary outcome is the proportion of persons with true referable DR attending the central ophthalmology clinic within eight weeks of screening. Secondary outcomes, by trial arm, include the proportion of persons attending the central ophthalmology clinic out of all those referred, sensitivity and specificity, the number of false positive referrals, acceptability and fidelity of AI-supported screening.

Ethics and dissemination

The LSHTM, Kilimanjaro Christian Medical Centre and Tanzanian National Institute of Medical Research ethics committees have approved the trial. The results will be submitted to peer-reviewed journals for publication.

Trial registration number: ISRCTN18317152

For peer review only

STRENGTHS AND LIMITATION OF THIS STUDY

- This is one of the first randomised controlled trials undertaken in Africa implementing an artificial intelligence (AI) system into an active clinical pathway
- Our primary outcome will measure the proportion of true referable DR cases attending the central ophthalmology clinic after referral from screening and will therefore measure the combined effect of AI-supported screening with a point-of-screening diagnosis and face-to-face counselling on attending follow-up
- The secondary outcomes will assess the acceptability of using this technology in this population as well as an economic evaluation which will inform implementation
- The eye department at the Kilimanjaro Christian Medical Centre (referral hospital) has a full range of ophthalmic services that is not representative of the wider African region
- For practical reasons, it is not possible to mask participants to their arm allocation

For peer review only

For peer review only - http://bmjopen.bmj.com/site/about/guidelines.xhtml

INTRODUCTION

By 2030, non-communicable diseases (NCD) are set to overtake communicable, maternal, neonatal, and nutritional diseases combined as the leading cause of mortality in sub-Saharan Africa (SSA).[1] The rising number of people with diabetes is a major contributor to this and from 2021 to 2045 the number of people with diabetes is projected to rise by 129% in SSA.[2] This is proportionally more than any other region of the world.[2,3]

Diabetic retinopathy (DR) is a common complication of diabetes and is a leading cause of blindness globally.[4] DR is asymptomatic in its early stages and often only begins to affect vision when the disease is at an advanced stage when is much less likely to respond to treatment; therefore screening is recommended to detect potentially sight-threatening DR and to refer people for assessment and treatment by an ophthalmologist. Early detection and timely treatment of sight threatening DR can prevent blindness in 95% of people with this stage of the disease.[5]

Several countries have successfully introduced DR screening programmes. For example, in the UK, following the introduction of a national DR screening programme, DR is no longer the leading cause of blindness in working-aged adults.[6] However, in many low- and middle-income countries (LMICs) there are no or very limited DR screening services and those that do exist suffer from several challenges.

At 12.3%, Tanzania is estimated to have the highest age-adjusted prevalence of diabetes in adults in Africa;[2] this has increased from an estimated prevalence of 2.3% in 2011.[2] In 2018 the Tanzanian Ministry of Health published guidelines which recommend annual screening of persons living with diabetes for DR.[7]

From 2010-2020 the Kilimanjaro Diabetic Programme (KDP) operated as a mobile community-based DR screening service across the Kilimanjaro region of Tanzania.[8] The screening team regularly visited each diabetic clinic and captured retinal photographs from people living with diabetes.. The photographs were then graded at a later date (usually after a few weeks) by an ophthalmology resident (specialist doctor in training) at the Kilimanjaro Christian Medical Centre (KCMC) Eye Department (the referral hospital). Patients screened were then contacted with their results via text message or phone call and were given advice on whether they needed to attend KCMC.

Major challenges were faced by the KDP as well as other similar programmes in the African region. First, there are not enough skilled eye care staff. Often the same eye care staff screen, follow-up and then treat patients with sight-threatening disease, placing additional pressure on already over stretched systems. The number of eye care staff currently in the African region is already unable to cope with the clinical demands and is very likely to be overwhelmed by the projected surge in demand for diabetic eye care services over the next 30 years. Second, there is limited quality control and training of staff in grading retinal images, and it is therefore unclear how accurate the DR gradings are within the current screening programmes. And third, in the KDP, it was noted that there were poor rates of follow-up (42%) for those referred with potentially sight threatening DR from screening to secondary eye care.[8] This meant the KDP was identifying people with potentially sight-threatening DR at risk of sight loss, but less than half of those people attended the central ophthalmology clinic for assessment; this strongly affected the ultimate impact of the programme.

In Tanzania there is no widely available postal service and particularly older, poorer people have no or limited access to telephones and the internet, making it practically impossible to provide some patients with their results and follow-up advice at a later date. Moreover, through 300 interviews with diabetic patients registered with the KDP, we found that an understanding that DR can be

For peer review only - http://bmjopen.bmj.com/site/about/guidelines.xhtml

treated and knowledge of the location of the referral hospital are positively related to adherence to follow-up. The provision of an immediate referral decision with real-time feedback and point-of-screening counselling is likely to improve the rates of follow-up for those referred to the eye clinic with sight-threatening DR.[9,10]

In view of the substantial projected increases in the number of people with diabetes worldwide, particularly in LMICs, there is an urgent need to test, develop and implement effective screening services for DR in order to prevent sight loss from the disease.

An alternative system to screen patients for DR that reduces demands on specialist staff, improves quality control and adherence to follow-up is needed. Advances in artificial intelligence (AI) for healthcare offer a potential alternative.

Ophthalmology is a leading medical specialty in healthcare AI, evidenced by the fact that the first autonomous medical AI device to get FDA approval (IDx-DR®) was for DR grading.[11] There are several AI systems commercially available for DR grading that have shown promisingly high sensitivities and specificities.[12] Such systems work by automating the interpretation of fundus photographs for DR, thereby reducing the need for trained clinical staff and, with high sensitivities and specificities, could enable the wide availability of high quality diagnostics within a short timeframe. Additionally, the immediate feedback provided by AI-supported screening software would enable a point-of-screening referral decision and counselling.

The current models of screening for DR do not work optimally in countries such as Tanzania. AI offers a promising solution to the specific problems faced in Tanzania, through decreased workforce pressures, improved quality control and potentially improved rates of follow-up for those people with sight threatening DR. However, in order for this technology to be appropriately and successfully implemented it needs to be tested and evaluated in prospective clinical trials.

AI-supported DR screening offers a potentially higher quality, lower unit cost and more accurate way to screen for DR, which is becoming an increasingly significant public health problem. This study will help determine whether this is the case and will provide an evidence base for the consideration of wider implementation.

Objectives

The primary objective of this study is to determine if AI-supported DR screening can increase the proportion of people with true referable DR that attend the central ophthalmology clinic following referral after screening for assessment and treatment. The secondary objectives are: (1) to compare prospective programmatic sensitivity and specificity of AI-supported and standard DR screening for the detection of referable DR and (2) to determine the impact of AI-supported screening on other key screening programme indicators including false positive referrals, treatment uptake, gradable versus ungradable image capture and to undertake an economic evaluation.

For peer review only - http://bmjopen.bmj.com/site/about/guidelines.xhtml

METHODS AND ANALYSIS

Trial Summary

In an individually randomised controlled trial, we will test the hypothesis that AI-supported DR screening increases the proportion of people with true referable DR who attend the specialist eye clinic for assessment and treatment following referral after screening.

Persons with diabetes attending screening for DR will be randomised to either the current practice DR screening pathway or the AI-supported DR screening pathway. The AI-supported screening arm will include automated retinal image analysis with an immediate referral decision and point-of-screening counselling. We will assess the proportion of people with true referable DR who attend a follow-up appointment at the central ophthalmology clinic within eight weeks of screening.

Figure 1; Overview of the trial

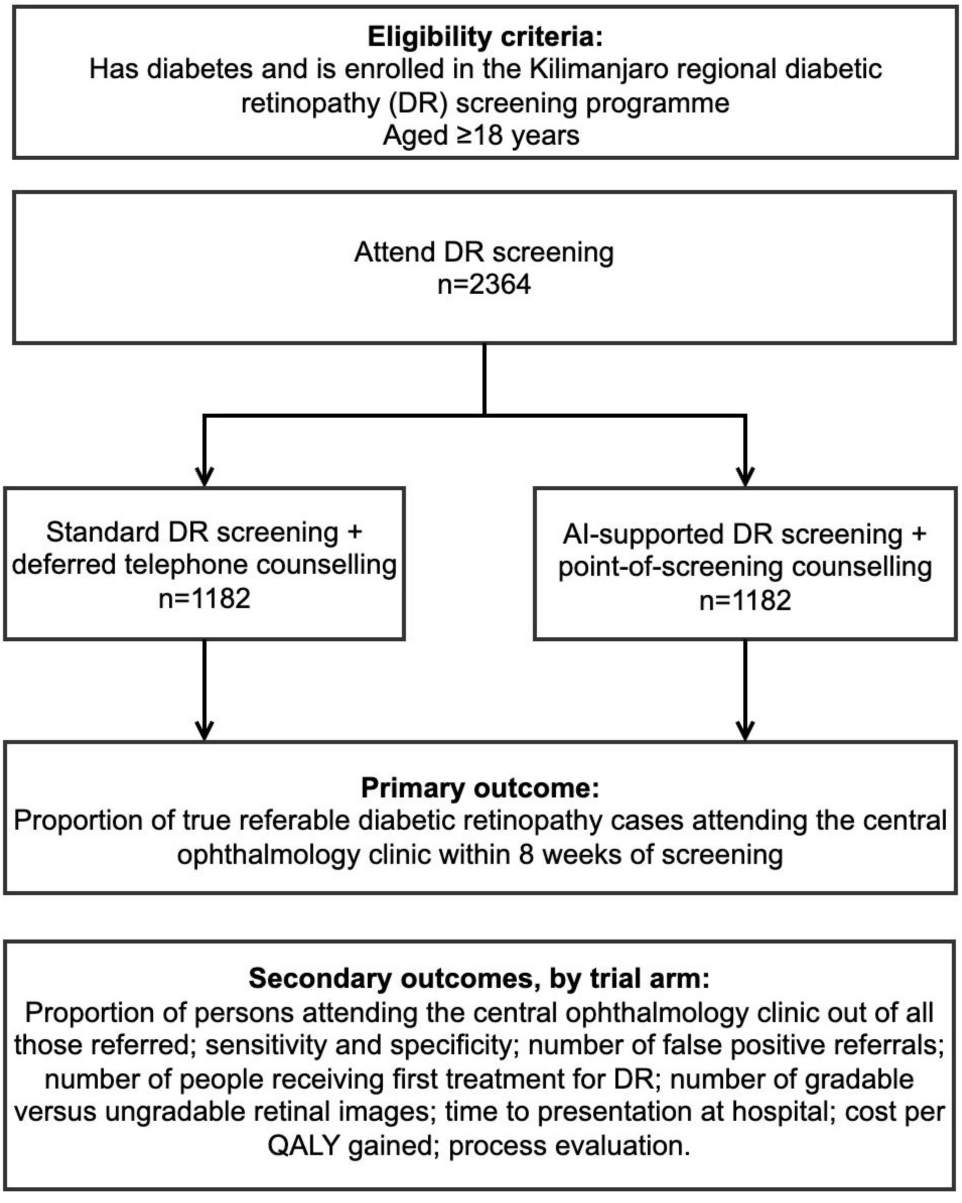

AI, artificial intelligence; QALY, quality adjusted life year

For peer review only - http://bmjopen.bmj.com/site/about/guidelines.xhtml

Trial Setting

The trial will be conducted in the Kilimanjaro region of northern Tanzania. KCMC Eye department is a tertiary referral centre with a full range of ophthalmic specialists including medical retina, vitreo-retinal, glaucoma, paediatric, cornea and cataract specialists. As well as fundus cameras, the department has OCT imaging and both retinal laser and anti-VEGF treatments for DR are available. KCMC Eye department is where patients will be referred after screening if they have referable DR.

Eligibility Criteria

Potential participants will need to meet the eligibility criteria stipulated in Table 1. In summary, potentially eligible participants include all persons aged over 18 years with diabetes in the Kilimanjaro region of Tanzania and who attend a DR screening event. The eligibility criteria are broad to reflect the reality of DR screening in Tanzania.

Table 1; Inclusion and exclusion criteria for enrolment into the randomised controlled trial

Inclusion criteria	Exclusions criteria (any of the following)
1. Adult (18 years and older) living with diabetes and attending diabetes clinics in Kilimanjaro region	1. Unable or unwilling to consent
2. Willing and able to give consent	2. Less than 18 years old
3. Agree to be randomised to either AI-supported or standard of care DR screening	

Informed consent

Any patients who may be eligible to participate will be given a participant information sheet and its contents will be read out to them by a study team member. They will be asked if they would be willing to participate and it will be explained to the potential participant that depending on which arm of the trial they are randomised to, they will either receive their result on the day of screening or some weeks later.

The patient will then have the opportunity to discuss any questions they might have. If the patient agrees to participate, they will be asked to read and sign or place a thumb print on the study consent form.

Clinical assessment

Following assessment of eligibility criteria and the informed consent process, the participant will proceed with the clinical assessment (Table 2). In summary, it will include a basic demographic and clinical history, visual acuity testing, blood sugar and blood pressure measurement followed by fundus photography.

Table 2; Demographic and clinical data

Assessment	Details
Demographic history	Gender, age, education level, occupation
Clinical history	Type of diabetes, duration of diabetes, current diabetes treatment (diet, medications, insulin), smoking history, known hypertension, previous heart attack or stroke
Visual acuity	Presenting best corrected VA. VA will be measured using a Tumbling-E logMAR chart with the PEEK VA smart phone application[13] by a trained study team member, for each eye separately.

For peer review only - http://bmjopen.bmj.com/site/about/guidelines.xhtml

Blood pressure	BP will be measured using a calibrated A&D (A&D Instruments Ltd, Abingdon, UK) UA-651 BP monitor.[14] Using the WHO STEPwise Approach to NCD risk factor Surveillance protocol,[15] three measurements will be taken one minute apart after the participant has rested for five minutes, with an average of the final two measurements recorded.
Random blood sugar	A random blood sugar measurement will be taken using a Contour Next Meter (Ascenia Diabetes Care Limited, UK)[16] and recorded in mmol/L.
Fundus photography	One drop of tropicamide 1% will be instilled into each eye separately. After 30 minutes (to allow adequate pupil dilation) a Topcon NW8 fundus camera will be used to capture two retinal photographs of each eye: one disc centred image and one macular centred image. The retinal images will be captured by a trained medical photographer.

VA = visual acuity; BP, blood pressure; NCD, non-communicable disease

Intervention and standard of care

Standard of care

The standard of care is the process by which patients have been screened for DR in the Kilimanjaro DR screening programme for >10 years. After retinal image capture, the photographs will be stored on a laptop and taken back to KCMC eye department. The images will be graded by an ophthalmology resident for the level of DR. Persons with referable DR will be contacted by text message or phone call some weeks after screening and advised to attend KCMC eye department for assessment with or without treatment. The counselling participants in the standard of care arm receive will follow the same format as the counselling given in the intervention arm of the trial, except that it will be delivered by telephone.

Intervention arm

The intervention in this trial is a complex intervention[17] including AI-supported retinal image analysis for DR grading with immediate feedback and referral advice followed by point-of-screening patient counselling.

There are two components to the intervention:

- AI retinal image analysis and DR grading
- Face-to-face counselling at the point of screening

The use of AI to grade retinal images for DR enables point-of-screening, face-to-face counselling as automated analysis provides immediate feedback after image submission, with a referral decision. The screening team member will inform the participant of their DR grade and proceed with the counselling component of the intervention. The point-of-screening counselling will include an explanation of what DR is, the potential to cause vision loss, the referral process and will emphasise that there are effective and safe treatments for DR.

AI system selection

There are several AI systems commercially available for DR screening.[12] A review process informed the choice of AI system in this trial.[18] In summary the following criteria were considered: existing regulatory approval (FDA approval or CE marking), ability to work offline, evidence of performance in an African population and the ability to work with different cameras. The SELENA+ AI software (EyRIS Pte Ltd)[19] was the only system to meet all these criteria and was therefore selected.

For peer review only

Counselling component of the intervention

In order to develop a clear, concise and contextually appropriate counselling script to be used to inform participants of their screening outcome, two focus group discussions (FGD) have been held. Participants in the FGDs included persons living with diabetes in the Kilimanjaro region and who are eligible to participate in the trial. A description of the research was provided and the participants were specifically asked what information they would like to be told when receiving their screening result. The counselling component of the intervention will be piloted with persons living with diabetes in the Kilimanjaro region prior to the start of the trial.

To ensure that the same information is shared in a similar form of words in both trial arms, the same counselling script will be used when patients are given their screening result irrespective of their allocation.

Randomisation

Randomisation sequence

A computer-generated randomisation list will be prepared by a statistician at London School of Hygiene & Tropical Medicine (LSHTM). The sequence will be in a 1:1 allocation ratio of standard DR screening to AI-supported DR screening, blocked with a random block size of 4 to 8.

Allocation concealment

The randomisation sequence will be concealed in sequentially numbered, opaque envelopes. The envelopes will be prepared by an administrator who is independent of all other aspects of the trial. A trained research team member will be responsible for opening the next envelope in numbered sequence and allocating the patient to either the AI-supported or standard of care DR screening pathway.

Masking

As immediate feedback is provided in the intervention arm versus no feedback at the point of screening in the standard of care arm, it will not be possible to mask participants or screening staff to the allocation. However, the arm the participant is allocated to will only be apparent after randomisation has occurred. The primary outcome is attendance at the central ophthalmology clinic within eight weeks of screening for those with true referrable DR; the research assistant collecting this information at KCMC eye clinic will be masked to the allocation.

Grading of retinal photographs

All retinal images captured in the trial will be graded by (1) the Tanzanian ophthalmology residents (2) the AI system and (3) UK certified graders. This will enable comparison of the performance of the AI system versus Tanzanian ophthalmology residents and against a reference standard (UK certified graders). All graders will be masked to the other DR gradings.

The grading of retinal photographs for DR will follow the UK National Screening Committee (NSC) guidelines.[20] This classification has historically been used in the KDP. Participants noted to have any of the following after grading by the UK certified graders will be defined as having true referable DR: pre-proliferative, proliferative DR, referable maculopathy or an ungradable retinal image. Referable maculopathy is defined as the presence of hard exudates or blot haemorrhage within 1 disc diameter of the fovea (centre of the retina). The worse eye will be used to assign participants overall DR grade.

Follow-up assessment

For peer review only - http://bmjopen.bmj.com/site/about/guidelines.xhtml

All participants screened in the trial will be tracked to determine whether or not they attend the specialist eye clinic. Any participant recommended to attend a follow-up appointment who has not done so within eight weeks of screening will be considered a non-attender.

For participants that attend the ophthalmology clinic we will document what, if any, treatment they are recommended and whether they commence the recommended treatment.

Lost to follow-up
As the primary outcome is attendance or not at the referral hospital within eight weeks of screening any persons not attending follow-up will be analysed as such.

Outcome measures

Primary outcome measure
The primary outcome will be the proportion of persons with true referable diabetic retinopathy who attend the central ophthalmology clinic within eight weeks of screening out of all those with true referable DR (defined by the reference standard), by trial arm.

All retinal images will be graded by UK certified graders to provide the reference standard. It will be these gradings that will determine which participants have true referable DR in each trial arm and this figure will be the denominator in our primary outcome analysis.

Secondary outcome measures
1. The proportion of persons that attend the central ophthalmology clinic within 8 weeks of screening out of all those referred, by trial arm
2. Sensitivity and specificity for grading referable DR
3. Number of false positive cases attending the central ophthalmology clinic, by trial arm
4. Number of patients receiving treatment after referral, by trial arm
5. Number of gradable versus ungradable retinal images
6. Time to presentation at hospital
7. Incremental cost per quality-adjusted life year (QALY) gained
8. Acceptability, appropriateness and fidelity of AI screening.

Data collection, management, confidentiality and access to data
Demographic and clinical data will be collected electronically in the field using a tablet (Huawei MediaPad T3; Model: KOB-WO9). The data collection forms will be developed using ODK software which is accessible via the tablet. ODK is a free open-source software fully encrypted for collecting, managing and using data is resource-constrained settings and works offline.[21] Data confidentiality will be maintained through restricted access to the data which will be password protected.

The retinal photographs collected in the study will be transferred onto an encrypted laptop after capture. Only authorized members of the study team will have access to these images. Any images transferred out of Tanzania for grading in the UK, in order to provide the reference standard, will be fully anonymised and labelled with internal linking IDs prior to transfer.

For peer review only - http://bmjopen.bmj.com/site/about/guidelines.xhtml

Data and safety monitoring board

The data and safety monitoring board (DSMB) for this trial comprises independent experts in statistics and ophthalmology appointed by the trial steering committee. The DSMB will meet bi-annually in person or virtually as needed to review the progress of the trial. Any modifications to the study protocol will be reviewed by the relevant ethics committees in the UK and Tanzania and the DSMB. The DSMB will also monitor any adverse events that occur during the trial.

Sample size considerations

This study is powered to test the hypothesis that AI-supported DR screening increases the proportion of persons with true referrable DR that attend the central ophthalmology clinic following referral after screening.

Our prior evaluation of the DR screening programme showed that the rate of follow-up for persons referred from screening to the ophthalmology clinic was 42%.[8] Using this value, and in discussion with our partners in Tanzania, it was agreed that a 33% increase in compliance with follow-up recommendations would be a significant and programmatically meaningful improvement to the service.

Assuming that 22.5% of persons screened have true referrable DR, with 1182 persons screened per trial arm (2364 persons in total), and therefore 266 persons with true referrable DR in each arm, we would have a 90% power to detect a 33% uplift (from 42% to 56%) in attendance at the ophthalmology clinic for those persons with true referable DR.

Analysis plan

The analysis will be by intention-to-treat (ITT) and all participant data will be analysed according to their randomisation allocation. However, not all participants randomised will be included in all analyses. For example, our primary outcome analysis includes only those persons with true referable DR as defined by the reference standard gradings.

The data will be analysed and reported according to the Consolidated Standards of Reporting Trials AI extension guidelines for reporting RCTs.[22] These guidelines are based on the CONSORT 2010 guidelines and have been adapted to produce a set of recommendations for clinical trials evaluating interventions with an AI component.

Primary outcome analysis: unadjusted analysis

The primary analysis will be a comparison of the proportion of persons with true referable DR out of all those with true referable DR (determined by the reference standard gradings) who attend the central ophthalmology clinic between the standard of care DR screening pathway (control) and the AI supported DR screening pathway (intervention). The primary analysis of the primary outcome will be by logistic regression model using attendance at eight weeks as the binary outcome, trial arm as the exposure and all participants who were classed as having referable DR by the reference standard measure will be included in the analysis. The intervention effect of AI-supported screening will be estimated as an odds ratio with a 95% confidence interval.

Primary outcome analysis: adjusted analysis

We will perform an adjusted analysis, adjusting for measured potential confounders including gender, education level and distance from the screening clinic to the referral hospital, to account for any

For peer review only - http://bmjopen.bmj.com/site/about/guidelines.xhtml

potential imbalance between the arms in terms of measured variables (or associated unmeasured confounders).

As attendance versus non-attendance at the specialist eye clinic for participants with true referable DR is the primary outcome measure, missing outcome data due to loss to follow-up will not be an issue. For example, if an individual enrolled in the trial is referred to the central ophthalmology clinic but does not attend the follow-up appointment, they will be considered a non-attender and will be included in the analysis as such; they will not be considered lost to follow-up.

Analysis of other potential determinants for attendance of true referrable diabetic retinopathy cases at the eye clinic

Logistic regression models will be used to analyse potential factors that may be associated with the primary outcome (attendance of true referable DR cases at the eye clinic). These potential factors include: age, gender, education level, occupation, distance from screening site to referral hospital, type of diabetic clinic (government funded, private, church-based), duration of diabetes, visual acuity, type of diabetic treatment (insulin, tablets) and any previous ophthalmic treatments.

We will initially perform a univariable analysis of all variables, followed by a multivariable analysis adjusting for all potential confounders. Both the univariable and multivariable analyses will be presented.

Secondary outcome analysis

The secondary outcomes will be analysed as detailed in Table 3. Binary outcomes will be analysed using logistic regression and continuous variables with linear regression. The sensitivity and specificity for referable DR of the AI system and the Tanzanian ophthalmology residents will be reported by arm and for all participants.

Table 3; Secondary outcome measures and analyses

**Secondary outcome measure**	**Analysis details**
Proportion of persons that attend the central ophthalmology clinic within 8 weeks of screening out of all those referred	We will measure this outcome, by trial arm, in the same manner as our primary outcome measure. This will be by logistic regression with attendance at 8 weeks the binary outcome and trial arm the exposure. However, the denominator for this outcome measure will be all persons referred in each trial arm, in contrast to only persons with true referable DR in our primary outcome analysis.
Sensitivity and specificity for grading referable DR	All retinal images collected in the study will be graded by the AI system, the Tanzanian ophthalmology residents and the UK certified graders (reference standard). This will enable us to calculate the sensitivity and specificity of both the AI system and the local Tanzanian ophthalmologists for grading referable DR, relative to the reference standard.
False positive cases attending the central ophthalmology clinic	The number of false positive cases attending the central ophthalmology clinic will be compared between trial arms by logistic regression, with false positive cases attending the ophthalmology clinic as the outcome measure and trial arm the exposure.
Number of patients receiving treatment after referral	The study is not powered to detect a difference in treatment uptake but we will undertake an exploratory analysis using logistic regression to compare

For peer review only

	treatment uptake between the trial arms for those participants that attend the ophthalmology clinic and who are recommended treatment.
Number of gradable versus ungradable retinal images	The number of images deemed ungradable will be compared between trials arms by logistic regression with gradable / ungradable retinal image as the outcome and trial arm the exposure.
Time to presentation at hospital	The time to presentation at hospital will be compared between trial arms for all those referred to the central ophthalmology clinic after screening. We will use Cox proportional hazards regression with trial arm as the primary predictor. Survival curves using Kaplan-Meier will be plotted for both trial arms to represent the time to presentation at hospital.
Incremental cost per QALY gained	The economic evaluation aims to assess the cost effectiveness of an AI supported screening model compared to standard of care model. Cost-effectiveness results will be expressed in terms of incremental cost-effectiveness ratios (ICERs) per QALY gained.<br><br>A Markov model will be built to undertake the analysis. Both deterministic and probabilistic sensitivity analyses will be performed to test the robustness of the model. Uncertainty in the parameters in the model will be reflected using probability distributions and the overall decision uncertainty will be presented using cost-effectiveness acceptability curves.
Acceptability, appropriateness and fidelity of AI screening	A process evaluation will be embedded within the trial and will be undertaken using the Medical Research Council guidance. Using semi-structured interviews with participants, screening team staff, ophthalmologists and Ministry of Health officials we will assess the acceptability of AI-supported DR screening from different perspectives (patients, staff, policy makers). These qualitative data will be analysed using Nvivo according to a condensation of meaning analysis and units of meaning will be coded into central themes.<br><br>The fidelity of AI-supported screening will be assessed during the trial by random spot checks evaluating the delivery of the intervention. Staff members will be asked to keep a log of any difficulties encountered whilst delivering the intervention, thereby highlighting implementation barriers and challenges.

DR, diabetic retinopathy. QALY, quality-adjusted life year.

Interim analysis

As there is negligible risk to participants an interim analysis is not required and data analysis will take place at the end of the study.

All retinal images collected during the study will be graded by local Tanzanian ophthalmology residents. Any images that are graded as referrable DR by the Tanzanian graders but were graded as non-referable by the AI (potential false negatives) will be reviewed by a senior grader. If the senior grader concludes that the participant does meet the referral threshold, participants will be contacted and advised to attend the eye clinic, thereby ensuring minimal risk to patients.

In such circumstances, the initial result from the AI grading will be used in the analysis and would therefore not affect the results.

For peer review only - http://bmjopen.bmj.com/site/about/guidelines.xhtml

Table 4; Trial Registration summary

Data Category	Information
Primary registry and trial indentifying no	ISRCTN Registry; ISRCTN18317152
Date of registration in primary registry	March 2nd 2023
Secondary identifying numbers	
Sources of monetary support	British Council for the Prevention of Blindness, CBM and the Sir Halley Stewart Trust
Primary sponsor	London School of Hygiene & Tropical Medicine
Secondary sponsor	
Contact for queries	Dr Charles Cleland; charles.cleland@lshtm.ac.uk
Title	Artificial intelligence support diabetic retinopathy screening in Tanzania
Countries of recruitment	Tanzania
Health condition(s) or problem(s) studies	Diabetic retinopathy
Intervention(s)	Artificial intelligence-supported retinal image analysis for diabetic retinopathy grading with immediate feedback and referral advice followed by point-of-screening patient counselling.
Key eligibility criteria	1. Adult (18 years and older) living with diabetes and attending a diabetic clinic<br>2. Agree to be randomised to either the AI-supported diabetic screening pathway or the standard of care diabetic retinopathy screening pathway
Study type	Randomised controlled trial
Date of first enrolment	March 8th 2023
Target sample size	2364
Recruitment status	Recruiting
Primary outcome(s)	Proportion of persons with true referable diabetic retinopathy who attend the central ophthalmology clinic within eight weeks of screening out of all those with true referable diabetic retinopathy
Key secondary outcomes	1. The proportion of persons that attend the central ophthalmology clinic within 8 weeks of screening out of all those referred, by trial arm<br>2. Sensitivity and specificity for grading referable DR<br>3. Number of false positive cases attending the central ophthalmology clinic, by trial arm<br>4. Number of patients receiving treatment after referral, by trial arm<br>5. Number of gradable versus ungradable retinal images<br>6. Time to presentation at hospital<br>7. Incremental cost per quality-adjusted life year (QALY) gained

Patient and public involvement

Our previous research highlighted the importance of knowledge of the referral process and the fact that DR can be treated as key factors that impact referral adherence,[23] both of which can be stated during point-of-screening counselling. We have held two focus group discussions with persons living with diabetes in the Kilimanjaro region, and who would be eligible for DR screening; the participants expressed a preference for a point-of-screening result over a delayed result.

For peer review only

Ethics and dissemination

Full ethical approval has been received from the London School of Hygiene & Tropical Medicine (LSHTM), UK and the Kilimanjaro Christian Medical Centre and the National Institute for Medical Research ethics committees in Tanzania. Any protocol modifications will be submitted to the relevant parties for review and / or approval. At the end of the study period any patients who still require treatment or follow-up will continue to be treated at KCMC Hospital. The trial has been registered with the ISRCTN clinical trials registry and the LSHTM is the trial sponsor. Data will be available upon request from the corresponding author on completion of the trial.The results of this trial will be submitted for publication in peer reviewed journals and will be presented at local and international meetings.

For peer review only

References

1. World Health Organization Regional Office of Africa. Non-communicable diseases. 2014. Available at: https://www.afro.who.int/health-topics/noncommunicable-diseases.
2. Federation ID. IDF Diabetes Atlas, 10th edn. Brussels, Belgium. 2021.
3. Atun R, Davies JI, Gale EAM, et al. Diabetes in sub-Saharan Africa: from clinical care to health policy. *Lancet Diabetes Endocrinol* 2017; **5**(8): 622-67.
4. Burton MJ, Ramke J, Marques AP, et al. The Lancet Global Health Commission on Global Eye Health: vision beyond 2020. *Lancet Glob Health* 2021; **9**(4): e489-e551.
5. Vujosevic S, Aldington SJ, Silva P, et al. Screening for diabetic retinopathy: new perspectives and challenges. *Lancet Diabetes Endocrinol* 2020; **8**(4): 337-47.
6. Scanlon PH. The English national screening programme for sight-threatening diabetic retinopathy. *J Med Screen* 2008; **15**(1): 1-4.
7. Bashir R SF, Peto T, Shilio B, Zondervan M. The Diabetic Eye Screening Programme in Tanzania — The VISION 2020 LINK between Dodoma and Belfast. *Eye News* 2021; **27**.
8. Cleland CR, Burton MJ, Hall C, et al. Diabetic retinopathy in Tanzania: prevalence and risk factors at entry into a regional screening programme. *Trop Med Int Health* 2016; **21**(3): 417-26.
9. Liu J, Gibson E, Ramchal S, et al. Diabetic Retinopathy Screening with Automated Retinal Image Analysis in a Primary Care Setting Improves Adherence to Ophthalmic Care. *Ophthalmol Retina* 2021; **5**(1): 71-7.
10. Mathenge W, Whitestone N, Nkurikiye J, et al. Impact of Artificial Intelligence Assessment of Diabetic Retinopathy on Referral Service Uptake in a Low-Resource Setting: The RAIDERS Randomized Trial. *Ophthalmology Science* 2022; **2**(4): 100168.
11. Abramoff MD, Lavin PT, Birch M, Shah N, Folk JC. Pivotal trial of an autonomous AI-based diagnostic system for detection of diabetic retinopathy in primary care offices. *NPJ Digit Med* 2018; **1**: 39.
12. Grzybowski A, Brona P, Lim G, et al. Artificial intelligence for diabetic retinopathy screening: a review. *Eye* 2020; **34**(3): 451-60.
13. Bastawrous A, Rono HK, Livingstone IAT, et al. Development and Validation of a Smartphone-Based Visual Acuity Test (Peek Acuity) for Clinical Practice and Community-Based Fieldwork. *JAMA Ophthalmology* 2015; **133**(8): 930-7.
14. Benetti E, Fania C, Palatini P. Validation of the A&D BP UA-651 device for home blood pressure measurement according to the European Society of Hypertension International Protocol revision 2010. *Blood Press Monit* 2014; **19**(1): 50-3.
15. World Health Organization. STEPwise approach to Noncommunicable Disease Surveillance (STEPS). 2017.
16. Harrison B, Leazenby C, Halldorsdottir S. Accuracy of the CONTOUR® blood glucose monitoring system. *J Diabetes Sci Technol* 2011; **5**(4): 1009-13.
17. Skivington K, Matthews L, Simpson SA, et al. Framework for the development and evaluation of complex interventions: gap analysis, workshop and consultation-informed update. *Health Technol Assess* 2021; **25**(57): 1-132.
18. Cleland CR BM, Evans J, Gordon I, Bascaran C. Artificial Intelligence for diabetic retinopathy in low- and middle-income countries: a scoping review. 2022.
19. EyRIS Pte Ltd. https://www.eyris.io.
20. Scanlon PH. The English National Screening Programme for diabetic retinopathy 2003-2016. *Acta diabetologica* 2017; **54**(6): 515-25.
21. Open Data Kit. https://getodk.org.
22. Liu X, Cruz Rivera S, Moher D, et al. Reporting guidelines for clinical trial reports for interventions involving artificial intelligence: the CONSORT-AI extension. *Nature Medicine* 2020; **26**(9): 1364-74.
23. Mtuya C, Cleland CR, Philippin H, et al. Reasons for poor follow-up of diabetic retinopathy patients after screening in Tanzania: a cross-sectional study. *BMC Ophthalmol* 2016; **16**: 115.

For peer review only

Author Statement: Searched the literature: CRC. Drafted initial protocol: CRC. Contributed to protocol development and revision: CRC, CB, WM, BS, FS, HP, APM, CE, AT, AD, PK, DM, MJB. Drafted this manuscript: CRC. Critically revised this manuscript: CRC, CB, WM, BS, FS, HP, APM, CE, AT, AD, PK, DM, MJB. Conceptualisation: CRC. Funding acquisition: CRC.

Funding: This research was supported by the British Council for the Prevention of Blindness, Christian Blind Mission and the Sir Halley Stewart Trust. MJB is supported by the Wellcome Trust (207472/Z/17/Z).

Disclaimer: The views expressed are those of the authors and not necessarily those of the NIHR or the Department of Health and Social Care. The funders have had, and will have, no role in the study design, data collection and analysis, decision to publish or preparation of the macuscript.

Competing interests: None declared.

Trial sponsor: London School of Hygiene & Tropical Medcine, Keppel Street, London. WC1E 7HT

Acknowledgments: The authors would like to thank the Kilimanjaro Christian Medical Centre Eye Department for their support with study co-ordination and implementation. The authors would like to thank the EyRIS PTE Ltd team for their support with providing the AI software, in particular Steven Ang. The authors would like to thank the staff at the Eye Screening Centre at the Homerton Hospital, in particular John Anderson and Louis Bolter, for their support with providing the reference standard gradings.

For peer review only

SPIRIT-AI

SPIRIT-AI Checklist: Recommended items to address in a protocol and related documents for clinical trials evaluating AI interventions

Section	SPIRIT 2013 Item[a]		SPIRIT-AI Item		Addressed on Page No [b]
Administrative Information					
Title	1	Descriptive title identifying the study design, population, interventions, and, if applicable, trial acronym	SPIRIT-AI 1(i) Elaboration	Indicate that the intervention involves artificial intelligence / machine learning and specify the type of model.	1
			SPIRIT-AI 1(ii) Elaboration	Specify the intended use of the AI intervention.	1
Trial registration	2a	Trial identifier and registry name. If not yet registered, name of intended registry			2
	2b	All items from the World Health Organization Trial Registration Data Set			14
Protocol version	3	Date and version identifier			Not relevant for protocol submitted for publication
Funding	4	Sources and types of financial, material, and other support			17
Roles and responsibilities	5a	Names, affiliations, and roles of protocol contributors			1 and 17
	5b	Name and contact information for the trial sponsor			17
	5c	Role of study sponsor and funders, if any, in study design; collection, management, analysis, and interpretation of data; writing of the report; and the decision to submit the report for publication, including whether they will have ultimate authority over any of these activities			17
	5d	Composition, roles, and responsibilities of the coordinating centre, steering committee, endpoint adjudication committee, data management team, and other individuals or groups overseeing the trial, if applicable (see Item 21a for data monitoring committee)			11
Introduction					
Background and rationale	6a	Description of research question and justification for undertaking the trial, including summary of relevant studies (published and unpublished) examining benefits and harms for each intervention	SPIRIT-AI 6a (i) Extension	Explain the intended use of the AI intervention in the context of the clinical pathway, including its purpose and its intended users (e.g. healthcare professionals, patients, public).	4-5 and 8-9

Cite as:

For peer review only

			SPIRIT-AI 6a (ii) Extension	Describe any pre-existing evidence for the AI intervention.	5
	6b	Explanation for choice of comparators			8
Objectives	7	Specific objectives or hypotheses			5
Trial design	8	Description of trial design including type of trial (eg, parallel group, crossover, factorial, single group), allocation ratio, and framework (eg, superiority, equivalence, noninferiority, exploratory)			6
Methods: Participants, Interventions and Outcomes					
Study setting	9	Description of study settings (eg, community clinic, academic hospital) and list of countries where data will be collected. Reference to where list of study sites can be obtained	SPIRIT-AI 9 Extension	Describe the onsite and offsite requirements needed to integrate the AI intervention into the trial setting.	7-8
Eligibility criteria	10	Inclusion and exclusion criteria for participants. If applicable, eligibility criteria for study centres and individuals who will perform the interventions (eg, surgeons, psychotherapists)	SPIRIT-AI 10 (i) Elaboration	State the inclusion and exclusion criteria at the level of participants.	7
			SPIRIT-AI 10 (ii) Extension	State the inclusion and exclusion criteria at the level of the input data.	7
Interventions	11a	Interventions for each group with sufficient detail to allow replication, including how and when they will be administered	SPIRIT-AI 11a (i) Extension	State which version of the AI algorithm will be used.	N/A – a commercially available AI system will be used.
			SPIRIT-AI 11a (ii) Extension	Specify the procedure for acquiring and selecting the input data for the AI intervention.	8
			SPIRIT-AI 11a (iii) Extension	Specify the procedure for assessing and handling poor quality or unavailable input data.	10
			SPIRIT-AI 11a (iv) Extension	Specify whether there is human-AI interaction in the handling of the input data, and what level of expertise is required for users.	8-9
			SPIRIT-AI 11a (v) Extension	Specify the output of the AI intervention.	8-9
			SPIRIT-AI 11a (vi) Extension	Explain the procedure for how the AI intervention's output will contribute to decision-making or other elements of clinical practice.	8-9
	11b	Criteria for discontinuing or modifying allocated interventions for a given trial participant (eg, drug dose change in response to harms, participant request, or improving/worsening disease)			13-14
	11c	Strategies to improve adherence to intervention protocols, and any procedures for monitoring adherence (eg, drug tablet return, laboratory tests)			8-9

Cite as:

SPIRIT-AI

	11d	Relevant concomitant care and interventions that are permitted or prohibited during the trial			N/A – not relevant to this study
Outcomes	12	Primary, secondary, and other outcomes, including the specific measurement variable (eg, systolic blood pressure), analysis metric (eg, change from baseline, final value, time to event), method of aggregation (eg, median, proportion), and time point for each outcome. Explanation of the clinical relevance of chosen efficacy and harm outcomes is strongly recommended			10
Participant timeline	13	Time schedule of enrolment, interventions (including any run-ins and washouts), assessments, and visits for participants. A schematic diagram is highly recommended (see Figure)			6-7
Sample size	14	Estimated number of participants needed to achieve study objectives and how it was determined, including clinical and statistical assumptions supporting any sample size calculations			11
Recruitment	15	Strategies for achieving adequate participant enrolment to reach target sample size			11
Methods: Assignment of Interventions (For Controlled Trials)					
Sequence generation	16A	Method of generating the allocation sequence (eg, computer-generated random numbers), and list of any factors for stratification. To reduce predictability of a random sequence, details of any planned restriction (eg, blocking) should be provided in a separate document that is unavailable to those who enrol participants or assign interventions			9
Allocation concealment mechanism	16b	Mechanism of implementing the allocation sequence (eg, central telephone; sequentially numbered, opaque, sealed envelopes), describing any steps to conceal the sequence until interventions are assigned			9
Implementation	16c	Who will generate the allocation sequence, who will enrol participants, and who will assign participants to interventions			9
Blinding (masking)	17a	Who will be blinded after assignment to interventions (eg, trial participants, care providers, outcome assessors, data analysts), and how			9
	17b	If blinded, circumstances under which unblinding is permissible, and procedure for revealing a participant's allocated intervention during the trial			N/A – single blinded study
Methods: Data Collection, Management, And Analysis					
Data collection methods	18a	Plans for assessment and collection of outcome, baseline, and other trial data, including any related processes to promote data quality (eg, duplicate measurements, training of assessors) and a description of study instruments (eg, questionnaires, laboratory tests) along with their reliability and validity, if known. Reference to where data collection forms can be found, if not in the protocol			10-11

Cite as:

For peer review only

SPIRIT-AI

	18b	Plans to promote participant retention and complete follow-up, including list of any outcome data to be collected for participants who discontinue or deviate from intervention protocols			10
Data management	19	Plans for data entry, coding, security, and storage, including any related processes to promote data quality (eg, double data entry; range checks for data values). Reference to where details of data management procedures can be found, if not in the protocol			10-11
Statistical methods	20a	Statistical methods for analysing primary and secondary outcomes. Reference to where other details of the statistical analysis plan can be found, if not in the protocol			11-13
	20b	Methods for any additional analyses (eg, subgroup and adjusted analyses)			11-13
	20c	Definition of analysis population relating to protocol non-adherence (eg, as randomised analysis), and any statistical methods to handle missing data (eg, multiple imputation)			11-13
Methods: Monitoring					
Data monitoring	21a	Composition of data monitoring committee (DMC); summary of its role and reporting structure; statement of whether it is independent from the sponsor and competing interests; and reference to where further details about its charter can be found, if not in the protocol. Alternatively, an explanation of why a DMC is not needed			11
	21b	Description of any interim analyses and stopping guidelines, including who will have access to these interim results and make the final decision to terminate the trial			13-14
Harms	22	Plans for collecting, assessing, reporting, and managing solicited and spontaneously reported adverse events and other unintended effects of trial interventions or trial conduct	SPIRIT-AI 22 Extension	Specify any plans to identify and analyse performance errors. If there are no plans for this, explain why not.	13-14
Auditing	23	Frequency and procedures for auditing trial conduct, if any, and whether the process will be independent from investigators and the sponsor			11
Ethics and Dissemination					
Research ethics approval	24	Plans for seeking research ethics committee/institutional review board (REC/IRB) approval			15
Protocol amendments	25	Plans for communicating important protocol modifications (eg, changes to eligibility criteria, outcomes, analyses) to relevant parties (eg, investigators, REC/IRBs, trial participants, trial registries, journals, regulators)			15
Consent or ascent	26a	Who will obtain informed consent or assent from potential trial participants or authorised surrogates, and how (see Item 32)			7

Cite as:

For peer review only - http://bmjopen.bmj.com/site/about/guidelines.xhtml

SPIRIT-AI

	26b	Additional consent provisions for collection and use of participant data and biological specimens in ancillary studies, if applicable			7
Confidentiality	27	How personal information about potential and enrolled participants will be collected, shared, and maintained in order to protect confidentiality before, during, and after the trial			10-11
Declaration of interests	28	Financial and other competing interests for principal investigators for the overall trial and each study site			17
Access to data	29	Statement of who will have access to the final trial dataset, and disclosure of contractual agreements that limit such access for investigators	SPIRIT-AI 29 Extension	State whether and how the AI intervention and/or its code can be accessed, including any restrictions to access or re-use.	N/A – a commercially available AI system will be used
Ancillary and post-trial care	30	Provisions, if any, for ancillary and post-trial care, and for compensation to those who suffer harm from trial participation			15
Dissemination policy	31a	Plans for investigators and sponsor to communicate trial results to participants, healthcare professionals, the public, and other relevant groups (eg, via publication, reporting in results databases, or other data sharing arrangements), including any publication restrictions			15
	31b	Authorship eligibility guidelines and any intended use of professional writers			17
	31c	Plans, if any, for granting public access to the full protocol, participant-level dataset, and statistical code			15
Appendices					
Informed consent materials	32	Model consent form and other related documentation given to participants and authorised surrogates			Appendix 1
Biological specimens	33	Plans for collection, laboratory evaluation, and storage of biological specimens for genetic or molecular analysis in the current trial and for future use in ancillary studies, if applicable			N/A – no biological specimens will be collected

[a] It is strongly recommended that this checklist be read in conjunction with the SPIRIT 2013 Explanation & Elaboration for important clarification on the items.
[b] Indicates page numbers to be completed by authors during protocol development.

Cite as:

For peer review only

BMJ Open

Artificial Intelligence-Supported Diabetic Retinopathy Screening in Tanzania: rationale and design of a randomised controlled trial

Journal:	*BMJ Open*
Manuscript ID	bmjopen-2023-075055.R1
Article Type:	Protocol
Date Submitted by the Author:	11-Dec-2023
Complete List of Authors:	Cleland, Charles; London School of Hygiene & Tropical Medicine, International Centre for Eye Health; Kilimanjaro Christian Medical Centre, Eye Department<br>Bascaran, Covadonga ; London School of Hygiene and Tropical Medicine International Centre for Eye Health,<br>Makupa, William; Kilimanjaro Christian Medical Centre, Eye Department<br>Shilio, Bernadetha; Union Government of Tanzania Ministry of Health Community Development Gender Elderly Children<br>Sandi, Frank; University of Dodoma School of Medicine and Nursing, Ophthalmology<br>Philippin, Heiko; London School of Hygiene & Tropical Medicine, International Centre for Eye Health; University of Freiburg Faculty of Medicine, Eye Centre<br>Marques, Ana Patricia; London School of Hygiene and Tropical Medicine International Centre for Eye Health,<br>Egan, Catherine; National Institute for Health and Care Research (NIHR) Biomedical Research Centre (BRC) for Ophthalmology, Moorfields Hospital London NHS Foundation Trust and Institute of Ophthalmology, University College London<br>Tufail, Adnan; National Institute for Health and Care Research (NIHR) Biomedical Research Centre (BRC) for Ophthalmology, Moorfields Hospital London NHS Foundation Trust and Institute of Ophthalmology, University College London<br>Keane, Pearse; National Institute for Health and Care Research (NIHR) Biomedical Research Centre (BRC) for Ophthalmology, Moorfields Hospital London NHS Foundation Trust and Institute of Ophthalmology, University College London<br>Denniston, Alastair; National Institute for Health and Care Research (NIHR) Biomedical Research Centre (BRC) for Ophthalmology, Moorfields Hospital London NHS Foundation Trust and Institute of Ophthalmology, University College London; National Institute for Health and Care Research, Birmingham Biomedical Research Centre<br>Macleod, David; London School of Hygiene & Tropical Medicine Department of Infectious Disease Epidemiology<br>Burton, Matthew J; London School of Hygiene & Tropical Medicine, International Centre for Eye Health; National Institute for Health and Care Research (NIHR) Biomedical Research Centre (BRC) for Ophthalmology, Moorfields Hospital London NHS Foundation Trust and Institute of Ophthalmology, University College London

**Primary Subject Heading**:	Ophthalmology
Secondary Subject Heading:	Global health, Health policy
Keywords:	Diabetic retinopathy < DIABETES & ENDOCRINOLOGY, PUBLIC HEALTH, Clinical Trial, OPHTHALMOLOGY

SCHOLARONE™ Manuscripts

Artificial Intelligence-Supported Diabetic Retinopathy Screening in Tanzania: rationale and design of a randomised controlled trial

Dr Charles R. Cleland[1,2], Dr Covadonga Bascaran[1], Dr William Makupa[2], Dr Bernadetha Shilio[3], Dr Frank Sandi[4], Dr Heiko Philippin[1,5], Dr Ana Patricia Marques[1], Dr Catherine Egan[6], Professor Adnan Tufail[6], Professor Pearse Keane[6], Professor Alastair K. Denniston[6,7,8], Dr David Macleod[9], Professor Matthew J. Burton[1,6]

[1] International Centre for Eye Health, London School of Hygiene and Tropical Medicine, London, UK

[2] Eye Department, Kilimanjaro Christian Medical Centre, Moshi, Tanzania

[3] Ministry of Health, Dodoma, Tanzania

[4] The University of Dodoma, Dodoma, Tanzania

[5] Eye Center, Medical Center, Faculty of Medicine, University of Freiburg, Germany

[6] National Institute for Health and Care Research (NIHR) Biomedical Research Centre (BRC) for Ophthalmology, Moorfields Hospital London NHS Foundation Trust and Institute of Ophthalmology, University College London, London, UK

[7] National Institute for Health and Care Research Birmingham Biomedical Research Centre, Birmingham, UK

[8] Academic Unit of Ophthalmology, Institute of Inflammation and Ageing, College of Medical and Dental Sciences, University of Birmingham, Birmingham, UK

[9] Department of Infectious Disease Epidemiology, Faculty of Epidemiology and Population Health, London School of Hygiene and Tropical Medicine, London, UK

Corresponding author: Dr Charles R Cleland

Address: International Centre for Eye Health, London School of Hygiene & Tropical Medicine, Keppel Street, London. WC1E 7HT

Email: charles.cleland@lshtm.ac.uk

Telephone number: +44 (0)20 7927 2329

For peer review only

ABSTRACT

Introduction

Globally diabetic retinopathy (DR) is a major cause of blindness. Sub-Saharan Africa is projected to see the largest proportionate increase in the number of people living with diabetes over the next two decades. Screening for DR is recommended to prevent sight loss; however, in many low- and middle-income countries, because of a lack of specialist eye care staff, current screening services for DR are not optimal. The use of artificial intelligence (AI) for DR screening, which automates the grading of retinal photographs and provides a point-of-screening result, offers an innovative potential solution to improve DR screening in Tanzania.

Methods and analysis

We will test the hypothesis that AI-supported DR screening increases the proportion of persons with true referable DR that attend the central ophthalmology clinic following referral after screening in a single-masked, parallel group individually randomised controlled trial. Participants will be randomised (1:1 ratio) to either AI-supported or the standard of care DR screening pathway. Participants allocated to the AI-supported screening pathway will receive their result followed by point-of-screening counselling immediately after retinal image capture. Participants in the standard of care arm will receive their result and counselling by phone once the retinal images have been graded in the usual way (typically after 2-4 weeks). The primary outcome is the proportion of persons with true referable DR attending the central ophthalmology clinic within eight weeks of screening. Secondary outcomes, by trial arm, include the proportion of persons attending the central ophthalmology clinic out of all those referred, sensitivity and specificity, the number of false positive referrals, acceptability and fidelity of AI-supported screening.

Ethics and dissemination

The LSHTM, Kilimanjaro Christian Medical Centre and Tanzanian National Institute of Medical Research ethics committees have approved the trial. The results will be submitted to peer-reviewed journals for publication.

Trial registration number: ISRCTN18317152

For peer review only

STRENGTHS AND LIMITATION OF THIS STUDY

- This study will measure the effect of artificial intelligence (AI)-supported diabetic retinopathy (DR) screening and face-to-face counselling on the proportion of persons with true referable DR that attend follow-up at the central ophthalmology clinic
- The study benefits from a pragmatic design with broad inclusion criteria and involves the implementation of an AI system into a DR screening programme
- For practical reasons, it is not possible to mask participants to their arm allocation
- This study is not powered to investigate whether AI-supported screening increases treatment uptake or improves visual outcomes

For peer review only

INTRODUCTION

By 2030, non-communicable diseases (NCD) are set to overtake communicable, maternal, neonatal, and nutritional diseases combined as the leading cause of mortality in sub-Saharan Africa (SSA).[1] The rising number of people with diabetes is a major contributor to this and from 2021 to 2045 the number of people with diabetes is projected to rise by 129% in SSA.[2] This is proportionally more than any other region of the world.[2, 3]

Diabetic retinopathy (DR) is a common complication of diabetes and is a leading cause of blindness globally.[4] DR is asymptomatic in its early stages and often only begins to affect vision when the disease is at an advanced stage when is much less likely to respond to treatment; therefore screening is recommended to detect potentially sight-threatening DR and to refer people for assessment and treatment by an ophthalmologist. Early detection and timely treatment of sight threatening DR can prevent blindness in 95% of people with this stage of the disease.[5]

Several countries have successfully introduced DR screening programmes. For example, in the UK, following the introduction of a national DR screening programme, DR is no longer the leading cause of blindness in working-aged adults.[6] However, in many low- and middle-income countries (LMICs) there are no or very limited DR screening services and those that do exist suffer from several challenges.

At 12.3%, Tanzania is estimated to have the highest age-adjusted prevalence of diabetes in adults in Africa;[2] this has increased from an estimated prevalence of 2.3% in 2011.[2] In 2018 the Tanzanian Ministry of Health published guidelines which recommend annual screening of persons living with diabetes for DR.[7]

From 2010-2020 the Kilimanjaro Diabetic Programme (KDP) operated as a mobile community-based DR screening service across the Kilimanjaro region of Tanzania.[8] The screening team regularly visited each diabetic clinic and captured retinal photographs from people living with diabetes. The photographs were then graded at a later date (usually after a few weeks) by an ophthalmology resident (specialist doctor in training) at the Kilimanjaro Christian Medical Centre (KCMC) Eye Department (the referral hospital). Patients screened were then contacted with their results via text message or phone call and were given advice on whether they needed to attend KCMC.

Major challenges were faced by the KDP as well as other similar programmes in the African region. First, there are not enough skilled eye care staff. Often the same eye care staff screen, follow-up and then treat patients with sight-threatening disease, placing additional pressure on already over stretched systems. The number of eye care staff currently in the African region is already unable to cope with the clinical demands and is very likely to be overwhelmed by the projected surge in demand for diabetic eye care services over the next 30 years. Second, there is limited quality control and training of staff in grading retinal images, and it is therefore unclear how accurate the DR gradings are within the current screening programmes. And third, in the KDP, it was noted that there were poor rates of follow-up (42%) for those referred with potentially sight threatening DR from screening to secondary eye care.[8] This meant the KDP was identifying people with potentially sight-threatening DR at risk of sight loss, but less than half of those people attended the central ophthalmology clinic for assessment; this strongly affected the ultimate impact of the programme.

In Tanzania there is no widely available postal service and particularly older, poorer people have no or limited access to telephones and the internet, making it practically impossible to provide some patients with their results and follow-up advice at a later date. Moreover, through 300 interviews with diabetic patients registered with the KDP, we found that an understanding that DR can be

For peer review only - http://bmjopen.bmj.com/site/about/guidelines.xhtml

treated and knowledge of the location of the referral hospital are positively related to adherence to follow-up. The provision of an immediate referral decision with real-time feedback and point-of-screening counselling is likely to improve the rates of follow-up for those referred to the eye clinic with sight-threatening DR.[9, 10]

In view of the substantial projected increases in the number of people with diabetes worldwide, particularly in LMICs, there is an urgent need to test, develop and implement effective screening services for DR in order to prevent sight loss from the disease.

An alternative system to screen patients for DR that reduces demands on specialist staff, improves quality control and adherence to follow-up is needed. Advances in artificial intelligence (AI) for healthcare offer a potential alternative.

Ophthalmology is a leading medical specialty in healthcare AI, evidenced by the fact that the first autonomous medical AI device to get FDA approval (IDx-DR®) was for DR grading.[11] There are several AI systems commercially available for DR grading that have shown promisingly high sensitivities and specificities.[12] Such systems work by automating the interpretation of fundus photographs for DR, thereby reducing the need for trained clinical staff and, with high sensitivities and specificities, could enable the wide availability of high quality diagnostics within a short timeframe. Additionally, the immediate feedback provided by AI-supported screening software would enable a point-of-screening referral decision and counselling.

The current models of screening for DR do not work optimally in countries such as Tanzania. AI offers a promising solution to the specific problems faced in Tanzania, through decreased workforce pressures, improved quality control and potentially improved rates of follow-up for those people with sight threatening DR. However, in order for this technology to be appropriately and successfully implemented it needs to be tested and evaluated in prospective clinical trials.

AI-supported DR screening offers a potentially higher quality, lower unit cost and more accurate way to screen for DR, which is becoming an increasingly significant public health problem. To our knowledge, this study is the first independent trial in SSA to measure whether the implementation of an AI system into a DR screening programme with a point-of-screening result and face-to-face counselling can increase the proportion of persons with true referable DR that attend the central ophthalmology clinic. The results will contribute valuable evidence for considering the wider implementation of AI technologies for DR screening.

Objectives

The primary objective of this study is to determine if AI-supported DR screening can increase the proportion of people with true referable DR that attend the central ophthalmology clinic following referral after screening for assessment and treatment. The secondary objectives are: (1) to compare prospective programmatic sensitivity and specificity of AI-supported and standard DR screening for the detection of referable DR and (2) to determine the impact of AI-supported screening on other key screening programme indicators including false positive referrals, treatment uptake, gradable versus ungradable image capture and to undertake an economic evaluation.

For peer review only

METHODS AND ANALYSIS

Trial Summary

In an individually randomised controlled trial, we will test the hypothesis that AI-supported DR screening increases the proportion of people with true referable DR who attend the specialist eye clinic for assessment and treatment following referral after screening.

Persons with diabetes attending screening for DR will be randomised to either the current practice DR screening pathway or the AI-supported DR screening pathway (Figure 1). The AI-supported screening arm will include automated retinal image analysis with an immediate referral decision and point-of-screening counselling. We will assess the proportion of people with true referable DR who attend a follow-up appointment at the central ophthalmology clinic within eight weeks of screening.

Figure 1; Overview of the trial

AI, artificial intelligence; QALY, quality adjusted life year

Trial Setting

The trial will be conducted in the Kilimanjaro and Arusha regions of northern Tanzania. KCMC Eye department is a tertiary referral centre with a full range of ophthalmic specialists including medical retina, vitreo-retinal, glaucoma, paediatric, cornea and cataract specialists. As well as fundus cameras, the department has OCT imaging and both retinal laser and anti-VEGF treatments for DR are available. KCMC Eye department is where patients will be referred after screening if they have referable DR.

Eligibility Criteria

Potential participants will need to meet the eligibility criteria stipulated in Table 1. In summary, potentially eligible participants include all persons aged over 18 years with diabetes in the Kilimanjaro or Arusha regions of Tanzania and who attend a diabetic clinic, which is where DR screening occurs. The eligibility criteria are broad to reflect the reality of DR screening in Tanzania.

Table 1; Inclusion and exclusion criteria for enrolment into the randomised controlled trial

Inclusion criteria	Exclusions criteria (any of the following)
1. Adult (18 years and older) living with diabetes and attending diabetes clinics in Kilimanjaro or Arusha regions	1. Unable or unwilling to consent
2. Willing and able to give consent	2. Less than 18 years old
3. Agree to be randomised to either AI-supported or standard of care DR screening	3. Already attending the central ophthalmology clinic or had a diabetic eye exam in the previous 12 months

Informed consent

Any patients who may be eligible to participate will be given a participant information sheet and its contents will be read out to them by a study team member. They will be asked if they would be willing to participate and it will be explained to the potential participant that depending on which arm of the trial they are randomised to, they will either receive their result on the day of screening or some weeks later.

For peer review only

The patient will then have the opportunity to discuss any questions they might have. If the patient agrees to participate, they will be asked to read and sign or place a thumb print on the study consent form.

Clinical assessment

Following assessment of eligibility criteria and the informed consent process, the participant will proceed with the clinical assessment (Table 2). In summary, it will include a basic demographic and clinical history, visual acuity testing, blood sugar and blood pressure measurement followed by fundus photography.

Table 2; Demographic and clinical data

Assessment	Details
Demographic history	Sex, age, education level, occupation, self-reported tribe / ethnicity, health insurance status
Clinical history	Type of diabetes, duration of diabetes, current diabetes treatment (diet, medications, insulin), smoking history, alcohol consumption, known hypertension, any known diabetes complications, previous heart attack or stroke,
Visual acuity	Presenting best corrected VA. VA will be measured using a Tumbling-E logMAR chart with the PEEK VA smart phone application[13] by a trained study team member, for each eye separately.
Blood pressure	BP will be measured using a calibrated A&D (A&D Instruments Ltd, Abingdon, UK) UA-651 BP monitor.[14] Using the WHO STEPwise Approach to NCD risk factor Surveillance protocol,[15] three measurements will be taken one minute apart after the participant has rested for five minutes, with an average of the final two measurements recorded.
Random blood sugar	A random blood sugar measurement will be taken using a Contour Next Meter (Ascenia Diabetes Care Limited, UK)[16] and recorded in mmol/L.
Fundus photography	One drop of tropicamide 1% will be instilled into each eye separately. After 30 minutes (to allow adequate pupil dilation) a Topcon NW8 fundus camera will be used to capture two retinal photographs of each eye: one disc centred image and one macular centred image. The retinal images will be captured by a trained medical photographer.

VA = visual acuity; BP, blood pressure; NCD, non-communicable disease

Intervention and standard of care

Standard of care

The standard of care is the process by which patients have been screened for DR in the Kilimanjaro DR screening programme for >10 years. After retinal image capture, the photographs will be stored on a laptop and taken back to KCMC eye department. The images will be graded by an ophthalmology resident for the level of DR. Persons with referable DR will be contacted by text message or phone call some weeks after screening and advised to attend KCMC eye department for assessment with or without treatment. The counselling participants in the standard of care arm receive will follow the same format as the counselling given in the intervention arm of the trial, except that it will be delivered by telephone.

Intervention arm

The intervention in this trial is a complex intervention[17] including AI-supported retinal image analysis for DR grading with immediate feedback and referral advice followed by point-of-screening patient counselling.

For peer review only - http://bmjopen.bmj.com/site/about/guidelines.xhtml

There are two components to the intervention:

- AI retinal image analysis and DR grading
- Face-to-face counselling at the point of screening

The use of AI to grade retinal images for DR enables point-of-screening, face-to-face counselling as automated analysis provides immediate feedback after image submission, with a referral decision. The screening team member will inform the participant of their DR grade and proceed with the counselling component of the intervention. The point-of-screening counselling will include an explanation of what DR is, the potential to cause vision loss, the referral process and will emphasise that there are effective and safe treatments for DR.

AI system selection

There are several AI systems commercially available for DR screening.[12] A review process informed the choice of AI system in this trial.[18] In summary the following criteria were considered: existing regulatory approval (FDA approval or CE marking), ability to work offline, evidence of performance in an African population and the ability to work with different cameras. The SELENA+ AI software (EyRIS Pte Ltd)[19] was the only system to meet all these criteria and was therefore selected. In a prior published validation study in Zambia the SELENA+ AI system had a sensitivity of 92.25% and a specificity of 89.04% for detecting referable DR.[20]

Counselling component of the intervention

In order to develop a clear, concise and contextually appropriate counselling script to be used to inform participants of their screening outcome, two focus group discussions (FGD) have been held. Participants in the FGDs included persons living with diabetes in the Kilimanjaro region and who are eligible to participate in the trial. A description of the research was provided and the participants were specifically asked what information they would like to be told when receiving their screening result. The counselling component of the intervention will be piloted with persons living with diabetes in the Kilimanjaro region prior to the start of the trial.

To ensure that the same information is shared in a similar form of words in both trial arms, the same counselling script will be used when patients are given their screening result irrespective of their allocation.

Randomisation

Randomisation sequence

A computer-generated randomisation list will be prepared by a statistician at London School of Hygiene & Tropical Medicine (LSHTM). The sequence will be in a 1:1 allocation ratio of standard DR screening to AI-supported DR screening, blocked with a random block size of 4 to 8.

Allocation concealment

The randomisation sequence will be concealed in sequentially numbered, opaque envelopes. The envelopes will be prepared by an administrator who is independent of all other aspects of the trial. A trained research team member will be responsible for opening the next envelope in numbered sequence and allocating the patient to either the AI-supported or standard of care DR screening pathway.

For peer review only - http://bmjopen.bmj.com/site/about/guidelines.xhtml

Masking

As immediate feedback is provided in the intervention arm versus no feedback at the point of screening in the standard of care arm, it will not be possible to mask participants or screening staff to the allocation. However, the arm the participant is allocated to will only be apparent after randomisation has occurred. The primary outcome is attendance at the central ophthalmology clinic within eight weeks of screening for those with true referrable DR; the research assistant collecting this information at KCMC eye clinic will be masked to the allocation.

Grading of retinal photographs

All retinal images captured in the trial will be graded by (1) the Tanzanian ophthalmology residents (2) the AI system and (3) UK certified graders. This will enable comparison of the performance of the AI system versus Tanzanian ophthalmology residents and against a reference standard (UK certified graders). All graders will be masked to the other DR gradings.

For the reference standard gradings, the UK certified graders will undertake feature based grading and the retinal images will be labelled using a software programme (OptoMize®, NEC Software Solutions, UK) commonly used in the English National Screening Programme. All images will be labelled by two experienced graders from the English National Screening Programme. The graders will only have access to anonymised retinal images with no patient metadata. Any disagreements will be adjudicated by a third senior grader. All graders involved are subject to the English Diabetic Eye Screening quality assurance process.

Participants noted to have any of the following after grading by the UK certified graders will be defined as having true referable DR: pre-proliferative, proliferative DR, referable maculopathy or an ungradable retinal image. Referable maculopathy is defined as the presence of hard exudates or blot haemorrhage within 1 disc diameter of the fovea (centre of the retina) or a circinate or group of exudates within the macula. This definition of referable DR and maculoapthy follows the Tanzanian National Guidelines for the Management of DR (published by the Tanzanian Ministry of Health) which is the nationally approved standard of care for Tanzania.

Follow-up assessment

All participants screened in the trial will be tracked to determine whether or not they attend the central ophthalmology clinic. Any participant recommended to attend a follow-up appointment who has not done so within eight weeks of screening will be considered a non-attender. A follow-up period of less than eight weeks could bias in favour of the intervention as some participants in the standard of care arm may not receive their screening result until three to four weeks after screening. As attendance at the central ophthalmology clinic beyond eight weeks becomes increasingly less attributable to the DR screening result, it is our view that attendance within eight weeks of screening is most appropriate.

For participants that attend the ophthalmology clinic we will document what, if any, treatment they are recommended and whether they commence the recommended treatment.

Lost to follow-up

As the primary outcome is attendance or not at the referral hospital within eight weeks of screening any persons not attending follow-up will be analysed as such.

For peer review only - http://bmjopen.bmj.com/site/about/guidelines.xhtml

Outcome measures

Primary outcome measure

The primary outcome will be the proportion of persons with true referable diabetic retinopathy who attend the central ophthalmology clinic within eight weeks of screening out of all those with true referable DR (defined by the reference standard), by trial arm.

All retinal images will be graded by UK certified graders to provide the reference standard. It will be these gradings that will determine which participants have true referable DR in each trial arm and this figure will be the denominator in our primary outcome analysis.

Secondary outcome measures

1. The proportion of persons that attend the central ophthalmology clinic within 8 weeks of screening out of all those referred, by trial arm
2. Sensitivity and specificity for grading referable DR
3. Number of false positive cases attending the central ophthalmology clinic, by trial arm
4. Number of patients receiving treatment after referral, by trial arm
5. Number of gradable versus ungradable retinal images
6. Time to presentation at hospital
7. Incremental cost per quality-adjusted life year (QALY) gained
8. Acceptability, appropriateness and fidelity of AI screening.

Data collection, management, confidentiality and access to data

Demographic and clinical data will be collected electronically in the field using a tablet (Huawei MediaPad T3; Model: KOB-WO9). The data collection forms will be developed using ODK software which is accessible via the tablet. ODK is a free open-source software fully encrypted for collecting, managing and using data is resource-constrained settings and works offline.[21] Data confidentiality will be maintained through restricted access to the data which will be password protected.

The retinal photographs collected in the study will be transferred onto an encrypted laptop after capture. Only authorized members of the study team will have access to these images. Any images transferred out of Tanzania for grading in the UK, in order to provide the reference standard, will be fully anonymised and labelled with internal linking IDs prior to transfer.

For peer review only

Data and safety monitoring board

The data and safety monitoring board (DSMB) for this trial comprises independent experts in statistics and ophthalmology appointed by the trial steering committee. The DSMB will meet bi-annually in person or virtually as needed to review the progress of the trial. Any modifications to the study protocol will be reviewed by the relevant ethics committees in the UK and Tanzania and the DSMB. The DSMB will also monitor any adverse events that occur during the trial.

Sample size considerations

This study is powered to test the hypothesis that AI-supported DR screening increases the proportion of persons with true referrable DR that attend the central ophthalmology clinic following referral after screening.

For peer review only - http://bmjopen.bmj.com/site/about/guidelines.xhtml

Our prior evaluation of the DR screening programme showed that the rate of follow-up for persons referred from screening to the ophthalmology clinic was 42%.[8] Using this value, and in discussion with our partners in Tanzania, it was agreed that a 33% increase in compliance with follow-up recommendations would be a significant and programmatically meaningful improvement to the service.

Assuming that 22.5% of persons screened have true referrable DR, with 1182 persons screened per trial arm (2364 persons in total), and therefore 266 persons with true referrable DR in each arm, we would have a 90% power to detect a 33% uplift (from 42% to 56%) in attendance at the ophthalmology clinic for those persons with true referable DR.

Analysis plan

The analysis will be by intention-to-treat (ITT) and all participant data will be analysed according to their randomisation allocation. However, not all participants randomised will be included in all analyses. For example, our primary outcome analysis includes only those persons with true referable DR as defined by the reference standard gradings.

The data will be analysed and reported according to the Consolidated Standards of Reporting Trials AI extension guidelines for reporting RCTs.[22] These guidelines are based on the CONSORT 2010 guidelines and have been adapted to produce a set of recommendations for clinical trials evaluating interventions with an AI component.

Primary outcome analysis: unadjusted analysis

The primary analysis will be a comparison of the proportion of persons with true referable DR out of all those with true referable DR (determined by the reference standard gradings) who attend the central ophthalmology clinic between the standard of care DR screening pathway (control) and the AI supported DR screening pathway (intervention). The primary analysis of the primary outcome will be by logistic regression model using attendance at eight weeks as the binary outcome, trial arm as the exposure and all participants who were classed as having referable DR by the reference standard measure will be included in the analysis. The intervention effect of AI-supported screening will be estimated as an odds ratio with a 95% confidence interval.

Primary outcome analysis: adjusted analysis

We will perform an adjusted analysis, adjusting for measured potential confounders including gender, education level and distance from the screening clinic to the referral hospital, to account for any potential imbalance between the arms in terms of measured variables (or associated unmeasured confounders).

As attendance versus non-attendance at the specialist eye clinic for participants with true referable DR is the primary outcome measure, missing outcome data due to loss to follow-up will not be an issue. For example, if an individual enrolled in the trial is referred to the central ophthalmology clinic but does not attend the follow-up appointment, they will be considered a non-attender and will be included in the analysis as such; they will not be considered lost to follow-up.

Analysis of other potential determinants for attendance of true referrable diabetic retinopathy cases at the eye clinic

For peer review only

Logistic regression models will be used to analyse potential factors that may be associated with the primary outcome (attendance of true referable DR cases at the eye clinic). These potential factors include: age, gender, education level, occupation, distance from screening site to referral hospital, type of diabetic clinic (government funded, private, church-based), duration of diabetes, visual acuity, type of diabetic treatment (insulin, tablets) and any previous ophthalmic treatments.

We will initially perform a univariable analysis of all variables, followed by a multivariable analysis adjusting for all potential confounders. Both the univariable and multivariable analyses will be presented.

Secondary outcome analysis
The secondary outcomes will be analysed as detailed in Table 3. Binary outcomes will be analysed using logistic regression and continuous variables with linear regression. The sensitivity and specificity for referable DR of the AI system and the Tanzanian ophthalmology residents will be reported by arm and for all participants.

For peer review only

Table 3; Secondary outcome measures and analyses

Secondary outcome measure	Analysis details
Proportion of persons that attend the central ophthalmology clinic within 8 weeks of screening out of all those referred	We will measure this outcome, by trial arm, in the same manner as our primary outcome measure. This will be by logistic regression with attendance at 8 weeks the binary outcome and trial arm the exposure. However, the denominator for this outcome measure will be all persons referred in each trial arm, in contrast to only persons with true referable DR in our primary outcome analysis.
Sensitivity and specificity for grading referable DR	All retinal images collected in the study will be graded by the AI system, the Tanzanian ophthalmology residents and the UK certified graders (reference standard). This will enable us to calculate the sensitivity and specificity of both the AI system and the local Tanzanian ophthalmologists for grading referable DR, relative to the reference standard.
False positive cases attending the central ophthalmology clinic	The number of false positive cases attending the central ophthalmology clinic will be compared between trial arms by logistic regression, with false positive cases attending the ophthalmology clinic as the outcome measure and trial arm the exposure.
Number of patients receiving treatment after referral	The study is not powered to detect a difference in treatment uptake but we will undertake an exploratory analysis using logistic regression to compare treatment uptake between the trial arms for those participants that attend the ophthalmology clinic and who are recommended treatment.
Number of gradable versus ungradable retinal images	The number of images deemed ungradable will be compared between trials arms by logistic regression with gradable / ungradable retinal image as the outcome and trial arm the exposure.
Time to presentation at hospital	The time to presentation at hospital will be compared between trial arms for all those referred to the central ophthalmology clinic after screening. We will use Cox proportional hazards regression with trial arm as the primary predictor. Survival curves using Kaplan-Meier will be plotted for both trial arms to represent the time to presentation at hospital.

For peer review only - http://bmjopen.bmj.com/site/about/guidelines.xhtml

Incremental cost per QALY gained	The economic evaluation aims to assess the cost effectiveness of an AI supported screening model compared to standard of care model. Cost-effectiveness results will be expressed in terms of incremental cost-effectiveness ratios (ICERs) per QALY gained.<br><br>A Markov model will be built to undertake the analysis. Both deterministic and probabilistic sensitivity analyses will be performed to test the robustness of the model. Uncertainty in the parameters in the model will be reflected using probability distributions and the overall decision uncertainty will be presented using cost-effectiveness acceptability curves.
Acceptability, appropriateness and fidelity of AI screening	A process evaluation will be embedded within the trial and will be undertaken using the Medical Research Council guidance.[23] Using semi-structured interviews with participants, screening team staff, ophthalmologists and Ministry of Health officials we will assess the acceptability of AI-supported DR screening from different perspectives (patients, staff, policy makers). These qualitative data will be analysed using Nvivo according to a condensation of meaning analysis and units of meaning will be coded into central themes.<br><br>The fidelity of AI-supported screening will be assessed during the trial by random spot checks evaluating the delivery of the intervention. Staff members will be asked to keep a log of any difficulties encountered whilst delivering the intervention, thereby highlighting implementation barriers and challenges.

DR, diabetic retinopathy. QALY, quality-adjusted life year.

Interim analysis

As there is negligible risk to participants an interim analysis is not required and data analysis will take place at the end of the study.

All retinal images collected during the study will be graded by local Tanzanian ophthalmology residents. Any images that are graded as referrable DR by the Tanzanian graders but were graded as non-referable by the AI (potential false negatives) will be reviewed by a senior grader. If the senior grader concludes that the participant does meet the referral threshold, participants will be contacted and advised to attend the eye clinic, thereby ensuring minimal risk to patients.

In such circumstances, the initial result from the AI grading will be used in the analysis and would therefore not affect the results.

Table 4; Trial Registration summary

**Data Category**	**Information**
Primary registry and trial indentifying no	ISRCTN Registry; ISRCTN18317152
Date of registration in primary registry	March 2nd 2023
Secondary identifying numbers	
Sources of monetary support	British Council for the Prevention of Blindness, CBM and the Sir Halley Stewart Trust
Primary sponsor	London School of Hygiene & Tropical Medicine
Secondary sponsor	

For peer review only - http://bmjopen.bmj.com/site/about/guidelines.xhtml

Contact for queries	Dr Charles Cleland; charles.cleland@lshtm.ac.uk
Title	Artificial intelligence support diabetic retinopathy screening in Tanzania
Countries of recruitment	Tanzania
Health condition(s) or problem(s) studies	Diabetic retinopathy
Intervention(s)	Artificial intelligence-supported retinal image analysis for diabetic retinopathy grading with immediate feedback and referral advice followed by point-of-screening patient counselling.
Key eligibility criteria	1. Adult (18 years and older) living with diabetes and attending a diabetic clinic<br>2. Agree to be randomised to either the AI-supported diabetic screening pathway or the standard of care diabetic retinopathy screening pathway
Study type	Randomised controlled trial
Date of first enrolment	March 8th 2023
Target sample size	2364
Recruitment status	Recruiting
Primary outcome(s)	Proportion of persons with true referable diabetic retinopathy who attend the central ophthalmology clinic within eight weeks of screening out of all those with true referable diabetic retinopathy
Key secondary outcomes	1. The proportion of persons that attend the central ophthalmology clinic within 8 weeks of screening out of all those referred, by trial arm<br>2. Sensitivity and specificity for grading referable DR<br>3. Number of false positive cases attending the central ophthalmology clinic, by trial arm<br>4. Number of patients receiving treatment after referral, by trial arm<br>5. Number of gradable versus ungradable retinal images<br>6. Time to presentation at hospital<br>7. Incremental cost per quality-adjusted life year (QALY) gained

Patient and public involvement

Our previous research highlighted the importance of knowledge of the referral process and the fact that DR can be treated as key factors that impact referral adherence,[24] both of which can be stated during point-of-screening counselling. We have held two focus group discussions with persons living with diabetes in the Kilimanjaro region, and who would be eligible for DR screening; the participants expressed a preference for a point-of-screening result over a delayed result.

Ethics and dissemination

Full ethical approval has been received from the London School of Hygiene & Tropical Medicine (LSHTM), UK and the Kilimanjaro Christian Medical Centre and the National Institute for Medical Research ethics committees in Tanzania. Any protocol modifications will be submitted to the relevant parties for review and / or approval. At the end of the study period any patients who still require treatment or follow-up will continue to be treated at KCMC Hospital. The trial has been registered with the ISRCTN clinical trials registry and the LSHTM is the trial sponsor (Table 4). Data will be available upon request from the corresponding author on completion of the trial.The results of this trial will be submitted for publication in peer reviewed journals and will be presented at local and international meetings.

For peer review only - http://bmjopen.bmj.com/site/about/guidelines.xhtml

References

1. *World Health Organization Regional Office of Africa. Non-communicable diseases. 2014. Available at: https://www.afro.who.int/health-topics/noncommunicable-diseases*.
2. Federation, I.D., *IDF Diabetes Atlas, 10th edn. Brussels, Belgium.* 2021.
3. Atun, R., et al., *Diabetes in sub-Saharan Africa: from clinical care to health policy.* Lancet Diabetes Endocrinol, 2017. **5**(8): p. 622-667.
4. Burton, M.J., et al., *The Lancet Global Health Commission on Global Eye Health: vision beyond 2020.* Lancet Glob Health, 2021. **9**(4): p. e489-e551.
5. Vujosevic, S., et al., *Screening for diabetic retinopathy: new perspectives and challenges.* Lancet Diabetes Endocrinol, 2020. **8**(4): p. 337-347.
6. Scanlon, P.H., *The English national screening programme for sight-threatening diabetic retinopathy.* J Med Screen, 2008. **15**(1): p. 1-4.
7. Bashir R, S.F., Peto T, Shilio B, Zondervan M, *The Diabetic Eye Screening Programme in Tanzania — The VISION 2020 LINK between Dodoma and Belfast.* Eye News, 2021. **27**.
8. Cleland, C.R., et al., *Diabetic retinopathy in Tanzania: prevalence and risk factors at entry into a regional screening programme.* Trop Med Int Health, 2016. **21**(3): p. 417-26.
9. Liu, J., et al., *Diabetic Retinopathy Screening with Automated Retinal Image Analysis in a Primary Care Setting Improves Adherence to Ophthalmic Care.* Ophthalmol Retina, 2021. **5**(1): p. 71-77.
10. Mathenge, W., et al., *Impact of Artificial Intelligence Assessment of Diabetic Retinopathy on Referral Service Uptake in a Low-Resource Setting: The RAIDERS Randomized Trial.* Ophthalmology Science, 2022. **2**(4): p. 100168.
11. Abramoff, M.D., et al., *Pivotal trial of an autonomous AI-based diagnostic system for detection of diabetic retinopathy in primary care offices.* NPJ Digit Med, 2018. **1**: p. 39.
12. Grzybowski, A., et al., *Artificial intelligence for diabetic retinopathy screening: a review.* Eye, 2020. **34**(3): p. 451-460.
13. Bastawrous, A., et al., *Development and Validation of a Smartphone-Based Visual Acuity Test (Peek Acuity) for Clinical Practice and Community-Based Fieldwork.* JAMA Ophthalmology, 2015. **133**(8): p. 930-937.
14. Benetti, E., C. Fania, and P. Palatini, *Validation of the A&D BP UA-651 device for home blood pressure measurement according to the European Society of Hypertension International Protocol revision 2010.* Blood Press Monit, 2014. **19**(1): p. 50-3.
15. *World Health Organization. STEPwise approach to Noncommunicable Disease Surveillance (STEPS).* 2017.
16. Harrison, B., C. Leazenby, and S. Halldorsdottir, *Accuracy of the CONTOUR® blood glucose monitoring system.* J Diabetes Sci Technol, 2011. **5**(4): p. 1009-13.
17. Skivington, K., et al., *Framework for the development and evaluation of complex interventions: gap analysis, workshop and consultation-informed update.* Health Technol Assess, 2021. **25**(57): p. 1-132.
18. Cleland, C.R., et al., *Artificial intelligence for diabetic retinopathy in low-income and middle-income countries: a scoping review.* BMJ Open Diabetes Res Care, 2023. **11**(4).
19. *EyRIS Pte Ltd*. Available from: https://www.eyris.io.
20. Bellemo, V., et al., *Artificial intelligence using deep learning to screen for referable and vision-threatening diabetic retinopathy in Africa: a clinical validation study.* Lancet Digit Health, 2019. **1**(1): p. e35-e44.

For peer review only - http://bmjopen.bmj.com/site/about/guidelines.xhtml

21. *Open Data Kit*. Available from: https://getodk.org.
22. Liu, X., et al., *Reporting guidelines for clinical trial reports for interventions involving artificial intelligence: the CONSORT-AI extension.* Nature Medicine, 2020. **26**(9): p. 1364-1374.
23. Moore, G.F., et al., *Process evaluation of complex interventions: Medical Research Council guidance.* Bmj, 2015. **350**: p. h1258.
24. Mtuya, C., et al., *Reasons for poor follow-up of diabetic retinopathy patients after screening in Tanzania: a cross-sectional study.* BMC Ophthalmol, 2016. **16**: p. 115.

For peer review only

For peer review only - http://bmjopen.bmj.com/site/about/guidelines.xhtml

Author Statement: Searched the literature: CRC. Drafted initial protocol: CRC. Contributed to protocol development and revision: CRC, CB, WM, BS, FS, HP, APM, CE, AT, AD, PK, DM, MJB. Drafted this manuscript: CRC. Critically revised this manuscript: CRC, CB, WM, BS, FS, HP, APM, CE, AT, AD, PK, DM, MJB. Conceptualisation: CRC. Funding acquisition: CRC.

Funding: This research was supported by the British Council for the Prevention of Blindness, Christian Blind Mission and the Sir Halley Stewart Trust. PK is supported by a Moorfields Eye Charity Career Development Award (R190028A) and a UK Research & Innovation Future Leaders Fellowship (MR/T019050/1). MJB is supported by the Wellcome Trust (207472/Z/17/Z).

Disclaimer: The views expressed are those of the authors and not necessarily those of the NIHR or the Department of Health and Social Care. The funders have had, and will have, no role in the study design, data collection and analysis, decision to publish or preparation of the macuscript.

Competing interests: CE has received fees from Heidelberg Engineering, Inozyme Pharma. AT has received fees from Annexon, Apellis, Bayer, Genentech, Iveric Bio, Novartis, Oxurion, and Roche.PK has acted as a consultant for Roche, Novartis, Boehringer-Ingleheim, Adecco, Bitfount and is an equity owner in Big Picture Medical. PK has received speaker fees from Novartis, Gyroscope, Bayer, Thea, Boehringer-Ingleheim, Apellis, Abbvie, Alimera, Roche, Genentech, Specsavers, Heidelberg Engineering, Topcon, and Santen. PK has received travel support from Bayer, Topcon, and Roche. PK has attended advisory boards for Boehringer-Ingleheim, RetinAI, Novartis, Apellis, Abbvie, and Roche. No other authors declare any conflicts.

Trial sponsor: London School of Hygiene & Tropical Medcine, Keppel Street, London. WC1E 7HT

Acknowledgments: The authors would like to thank the Kilimanjaro Christian Medical Centre Eye Department for their support with study co-ordination and implementation. The authors would like to thank the EyRIS PTE Ltd team for their support with providing the AI software, in particular Steven Ang. The authors would like to thank the staff at the Eye Screening Centre at the Homerton Hospital, in particular John Anderson and Louis Bolter, for their support with providing the reference standard gradings.

For peer review only

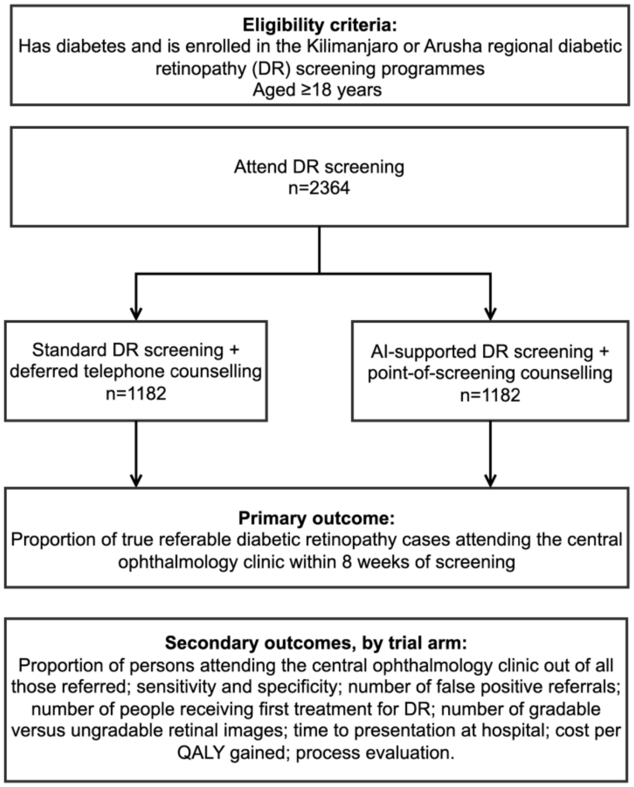

AI, artificial intelligence; QALY, quality adjusted life year

110x156mm (600 x 600 DPI)

SPIRIT-AI

SPIRIT-AI Checklist: Recommended items to address in a protocol and related documents for clinical trials evaluating AI interventions

Section	SPIRIT 2013 Item[a]		SPIRIT-AI Item		Addressed on Page No [b]
**Administrative Information**					
Title	1	Descriptive title identifying the study design, population, interventions, and, if applicable, trial acronym	SPIRIT-AI 1(i) Elaboration	Indicate that the intervention involves artificial intelligence / machine learning and specify the type of model.	1
			SPIRIT-AI 1(ii) Elaboration	Specify the intended use of the AI intervention.	1
Trial registration	2a	Trial identifier and registry name. If not yet registered, name of intended registry			2
	2b	All items from the World Health Organization Trial Registration Data Set			14
Protocol version	3	Date and version identifier			Not relevant for protocol submitted for publication
Funding	4	Sources and types of financial, material, and other support			17
Roles and responsibilities	5a	Names, affiliations, and roles of protocol contributors			1 and 17
	5b	Name and contact information for the trial sponsor			17
	5c	Role of study sponsor and funders, if any, in study design; collection, management, analysis, and interpretation of data; writing of the report; and the decision to submit the report for publication, including whether they will have ultimate authority over any of these activities			17
	5d	Composition, roles, and responsibilities of the coordinating centre, steering committee, endpoint adjudication committee, data management team, and other individuals or groups overseeing the trial, if applicable (see Item 21a for data monitoring committee)			11
**Introduction**					
Background and rationale	6a	Description of research question and justification for undertaking the trial, including summary of relevant studies (published and unpublished) examining benefits and harms for each intervention	SPIRIT-AI 6a (i) Extension	Explain the intended use of the AI intervention in the context of the clinical pathway, including its purpose and its intended users (e.g. healthcare professionals, patients, public).	4-5 and 8-9

Cite as:

For peer review only

SPIRIT-AI

			SPIRIT-AI 6a (ii) Extension	Describe any pre-existing evidence for the AI intervention.	5
	6b	Explanation for choice of comparators			8
Objectives	7	Specific objectives or hypotheses			5
Trial design	8	Description of trial design including type of trial (eg, parallel group, crossover, factorial, single group), allocation ratio, and framework (eg, superiority, equivalence, noninferiority, exploratory)			6
**Methods: Participants, Interventions and Outcomes**					
Study setting	9	Description of study settings (eg, community clinic, academic hospital) and list of countries where data will be collected. Reference to where list of study sites can be obtained	SPIRIT-AI 9 Extension	Describe the onsite and offsite requirements needed to integrate the AI intervention into the trial setting.	7-8
Eligibility criteria	10	Inclusion and exclusion criteria for participants. If applicable, eligibility criteria for study centres and individuals who will perform the interventions (eg, surgeons, psychotherapists)	SPIRIT-AI 10 (i) Elaboration	State the inclusion and exclusion criteria at the level of participants.	7
			SPIRIT-AI 10 (ii) Extension	State the inclusion and exclusion criteria at the level of the input data.	7
Interventions	11a	Interventions for each group with sufficient detail to allow replication, including how and when they will be administered	SPIRIT-AI 11a (i) Extension	State which version of the AI algorithm will be used.	N/A – a commercially available AI system will be used.
			SPIRIT-AI 11a (ii) Extension	Specify the procedure for acquiring and selecting the input data for the AI intervention.	8
			SPIRIT-AI 11a (iii) Extension	Specify the procedure for assessing and handling poor quality or unavailable input data.	10
			SPIRIT-AI 11a (iv) Extension	Specify whether there is human-AI interaction in the handling of the input data, and what level of expertise is required for users.	8-9
			SPIRIT-AI 11a (v) Extension	Specify the output of the AI intervention.	8-9
			SPIRIT-AI 11a (vi) Extension	Explain the procedure for how the AI intervention's output will contribute to decision-making or other elements of clinical practice.	8-9
	11b	Criteria for discontinuing or modifying allocated interventions for a given trial participant (eg, drug dose change in response to harms, participant request, or improving/worsening disease)			13-14
	11c	Strategies to improve adherence to intervention protocols, and any procedures for monitoring adherence (eg, drug tablet return, laboratory tests)			8-9

Cite as:

	11d	Relevant concomitant care and interventions that are permitted or prohibited during the trial			N/A – not relevant to this study
Outcomes	12	Primary, secondary, and other outcomes, including the specific measurement variable (eg, systolic blood pressure), analysis metric (eg, change from baseline, final value, time to event), method of aggregation (eg, median, proportion), and time point for each outcome. Explanation of the clinical relevance of chosen efficacy and harm outcomes is strongly recommended			10
Participant timeline	13	Time schedule of enrolment, interventions (including any run-ins and washouts), assessments, and visits for participants. A schematic diagram is highly recommended (see Figure)			6-7
Sample size	14	Estimated number of participants needed to achieve study objectives and how it was determined, including clinical and statistical assumptions supporting any sample size calculations			11
Recruitment	15	Strategies for achieving adequate participant enrolment to reach target sample size			11
Methods: Assignment of Interventions (For Controlled Trials)					
Sequence generation	16A	Method of generating the allocation sequence (eg, computer-generated random numbers), and list of any factors for stratification. To reduce predictability of a random sequence, details of any planned restriction (eg, blocking) should be provided in a separate document that is unavailable to those who enrol participants or assign interventions			9
Allocation concealment mechanism	16b	Mechanism of implementing the allocation sequence (eg, central telephone; sequentially numbered, opaque, sealed envelopes), describing any steps to conceal the sequence until interventions are assigned			9
Implementation	16c	Who will generate the allocation sequence, who will enrol participants, and who will assign participants to interventions			9
Blinding (masking)	17a	Who will be blinded after assignment to interventions (eg, trial participants, care providers, outcome assessors, data analysts), and how			9
	17b	If blinded, circumstances under which unblinding is permissible, and procedure for revealing a participant's allocated intervention during the trial			N/A – single blinded study
Methods: Data Collection, Management, And Analysis					
Data collection methods	18a	Plans for assessment and collection of outcome, baseline, and other trial data, including any related processes to promote data quality (eg, duplicate measurements, training of assessors) and a description of study instruments (eg, questionnaires, laboratory tests) along with their reliability and validity, if known. Reference to where data collection forms can be found, if not in the protocol			10-11

Cite as:

For peer review only - http://bmjopen.bmj.com/site/about/guidelines.xhtml

	18b	Plans to promote participant retention and complete follow-up, including list of any outcome data to be collected for participants who discontinue or deviate from intervention protocols			10
Data management	19	Plans for data entry, coding, security, and storage, including any related processes to promote data quality (eg, double data entry; range checks for data values). Reference to where details of data management procedures can be found, if not in the protocol			10-11
Statistical methods	20a	Statistical methods for analysing primary and secondary outcomes. Reference to where other details of the statistical analysis plan can be found, if not in the protocol			11-13
	20b	Methods for any additional analyses (eg, subgroup and adjusted analyses)			11-13
	20c	Definition of analysis population relating to protocol non-adherence (eg, as randomised analysis), and any statistical methods to handle missing data (eg, multiple imputation)			11-13
Methods: Monitoring					
Data monitoring	21a	Composition of data monitoring committee (DMC); summary of its role and reporting structure; statement of whether it is independent from the sponsor and competing interests; and reference to where further details about its charter can be found, if not in the protocol. Alternatively, an explanation of why a DMC is not needed			11
	21b	Description of any interim analyses and stopping guidelines, including who will have access to these interim results and make the final decision to terminate the trial			13-14
Harms	22	Plans for collecting, assessing, reporting, and managing solicited and spontaneously reported adverse events and other unintended effects of trial interventions or trial conduct	SPIRIT-AI 22 Extension	Specify any plans to identify and analyse performance errors. If there are no plans for this, explain why not.	13-14
Auditing	23	Frequency and procedures for auditing trial conduct, if any, and whether the process will be independent from investigators and the sponsor			11
Ethics and Dissemination					
Research ethics approval	24	Plans for seeking research ethics committee/institutional review board (REC/IRB) approval			15
Protocol amendments	25	Plans for communicating important protocol modifications (eg, changes to eligibility criteria, outcomes, analyses) to relevant parties (eg, investigators, REC/IRBs, trial participants, trial registries, journals, regulators)			15
Consent or ascent	26a	Who will obtain informed consent or assent from potential trial participants or authorised surrogates, and how (see Item 32)			7

Cite as:

SPIRIT-AI

	26b	Additional consent provisions for collection and use of participant data and biological specimens in ancillary studies, if applicable			7
Confidentiality	27	How personal information about potential and enrolled participants will be collected, shared, and maintained in order to protect confidentiality before, during, and after the trial			10-11
Declaration of interests	28	Financial and other competing interests for principal investigators for the overall trial and each study site			17
Access to data	29	Statement of who will have access to the final trial dataset, and disclosure of contractual agreements that limit such access for investigators	SPIRIT-AI 29 Extension	State whether and how the AI intervention and/or its code can be accessed, including any restrictions to access or re-use.	N/A – a commercially available AI system will be used
Ancillary and post-trial care	30	Provisions, if any, for ancillary and post-trial care, and for compensation to those who suffer harm from trial participation			15
Dissemination policy	31a	Plans for investigators and sponsor to communicate trial results to participants, healthcare professionals, the public, and other relevant groups (eg, via publication, reporting in results databases, or other data sharing arrangements), including any publication restrictions			15
	31b	Authorship eligibility guidelines and any intended use of professional writers			17
	31c	Plans, if any, for granting public access to the full protocol, participant-level dataset, and statistical code			15
Appendices					
Informed consent materials	32	Model consent form and other related documentation given to participants and authorised surrogates			Appendix 1
Biological specimens	33	Plans for collection, laboratory evaluation, and storage of biological specimens for genetic or molecular analysis in the current trial and for future use in ancillary studies, if applicable			N/A – no biological specimens will be collected

[a] It is strongly recommended that this checklist be read in conjunction with the SPIRIT 2013 Explanation & Elaboration for important clarification on the items.
[b] Indicates page numbers to be completed by authors during protocol development.

Cite as:

For peer review only

BMJ Open

Artificial Intelligence-Supported Diabetic Retinopathy Screening in Tanzania: rationale and design of a randomised controlled trial

Journal:	*BMJ Open*
Manuscript ID	bmjopen-2023-075055.R2
Article Type:	Protocol
Date Submitted by the Author:	12-Jan-2024
Complete List of Authors:	Cleland, Charles; London School of Hygiene & Tropical Medicine, International Centre for Eye Health; Kilimanjaro Christian Medical Centre, Eye Department<br>Bascaran, Covadonga ; London School of Hygiene and Tropical Medicine International Centre for Eye Health,<br>Makupa, William; Kilimanjaro Christian Medical Centre, Eye Department<br>Shilio, Bernadetha; Union Government of Tanzania Ministry of Health Community Development Gender Elderly Children<br>Sandi, Frank; University of Dodoma School of Medicine and Nursing, Ophthalmology<br>Philippin, Heiko; London School of Hygiene & Tropical Medicine, International Centre for Eye Health; University of Freiburg Faculty of Medicine, Eye Centre<br>Marques, Ana Patricia; London School of Hygiene and Tropical Medicine International Centre for Eye Health,<br>Egan, Catherine; National Institute for Health and Care Research (NIHR) Biomedical Research Centre (BRC) for Ophthalmology, Moorfields Hospital London NHS Foundation Trust and Institute of Ophthalmology, University College London<br>Tufail, Adnan; National Institute for Health and Care Research (NIHR) Biomedical Research Centre (BRC) for Ophthalmology, Moorfields Hospital London NHS Foundation Trust and Institute of Ophthalmology, University College London<br>Keane, Pearse; National Institute for Health and Care Research (NIHR) Biomedical Research Centre (BRC) for Ophthalmology, Moorfields Hospital London NHS Foundation Trust and Institute of Ophthalmology, University College London<br>Denniston, Alastair; National Institute for Health and Care Research (NIHR) Biomedical Research Centre (BRC) for Ophthalmology, Moorfields Hospital London NHS Foundation Trust and Institute of Ophthalmology, University College London; National Institute for Health and Care Research, Birmingham Biomedical Research Centre<br>Macleod, David; London School of Hygiene & Tropical Medicine Department of Infectious Disease Epidemiology<br>Burton, Matthew J; London School of Hygiene & Tropical Medicine, International Centre for Eye Health; National Institute for Health and Care Research (NIHR) Biomedical Research Centre (BRC) for Ophthalmology, Moorfields Hospital London NHS Foundation Trust and Institute of Ophthalmology, University College London

**Primary Subject Heading**:	Ophthalmology
Secondary Subject Heading:	Global health, Health policy
Keywords:	Diabetic retinopathy < DIABETES & ENDOCRINOLOGY, PUBLIC HEALTH, Clinical Trial, OPHTHALMOLOGY

SCHOLARONE™ Manuscripts

Artificial Intelligence-Supported Diabetic Retinopathy Screening in Tanzania: rationale and design of a randomised controlled trial

Dr Charles R. Cleland[1,2], Dr Covadonga Bascaran[1], Dr William Makupa[2], Dr Bernadetha Shilio[3], Dr Frank Sandi[4], Dr Heiko Philippin[1,5], Dr Ana Patricia Marques[1], Dr Catherine Egan[6], Professor Adnan Tufail[6], Professor Pearse Keane[6], Professor Alastair K. Denniston[6,7,8], Dr David Macleod[9], Professor Matthew J. Burton[1,6]

[1] International Centre for Eye Health, London School of Hygiene and Tropical Medicine, London, UK

[2] Eye Department, Kilimanjaro Christian Medical Centre, Moshi, Tanzania

[3] Ministry of Health, Dodoma, Tanzania

[4] The University of Dodoma, Dodoma, Tanzania

[5] Eye Center, Medical Center, Faculty of Medicine, University of Freiburg, Germany

[6] National Institute for Health and Care Research (NIHR) Biomedical Research Centre (BRC) for Ophthalmology, Moorfields Hospital London NHS Foundation Trust and Institute of Ophthalmology, University College London, London, UK

[7] National Institute for Health and Care Research Birmingham Biomedical Research Centre, Birmingham, UK

[8] Academic Unit of Ophthalmology, Institute of Inflammation and Ageing, College of Medical and Dental Sciences, University of Birmingham, Birmingham, UK

[9] Department of Infectious Disease Epidemiology, Faculty of Epidemiology and Population Health, London School of Hygiene and Tropical Medicine, London, UK

Corresponding author: Dr Charles R Cleland

Address: International Centre for Eye Health, London School of Hygiene & Tropical Medicine, Keppel Street, London. WC1E 7HT

Email: charles.cleland@lshtm.ac.uk

Telephone number: +44 (0)20 7927 2329

For peer review only

ABSTRACT

Introduction

Globally diabetic retinopathy (DR) is a major cause of blindness. Sub-Saharan Africa is projected to see the largest proportionate increase in the number of people living with diabetes over the next two decades. Screening for DR is recommended to prevent sight loss; however, in many low- and middle-income countries, because of a lack of specialist eye care staff, current screening services for DR are not optimal. The use of artificial intelligence (AI) for DR screening, which automates the grading of retinal photographs and provides a point-of-screening result, offers an innovative potential solution to improve DR screening in Tanzania.

Methods and analysis

We will test the hypothesis that AI-supported DR screening increases the proportion of persons with true referable DR that attend the central ophthalmology clinic following referral after screening in a single-masked, parallel group individually randomised controlled trial. Participants (2364) will be randomised (1:1 ratio) to either AI-supported or the standard of care DR screening pathway. Participants allocated to the AI-supported screening pathway will receive their result followed by point-of-screening counselling immediately after retinal image capture. Participants in the standard of care arm will receive their result and counselling by phone once the retinal images have been graded in the usual way (typically after 2-4 weeks). The primary outcome is the proportion of persons with true referable DR attending the central ophthalmology clinic within eight weeks of screening. Secondary outcomes, by trial arm, include the proportion of persons attending the central ophthalmology clinic out of all those referred, sensitivity and specificity, number of false positive referrals, acceptability and fidelity of AI-supported screening.

Ethics and dissemination

The LSHTM, Kilimanjaro Christian Medical Centre and Tanzanian National Institute of Medical Research ethics committees have approved the trial. The results will be submitted to peer-reviewed journals for publication.

Trial registration number: ISRCTN18317152

For peer review only

For peer review only - http://bmjopen.bmj.com/site/about/guidelines.xhtml

STRENGTHS AND LIMITATION OF THIS STUDY

- This study will measure the effect of artificial intelligence (AI)-supported diabetic retinopathy (DR) screening and face-to-face counselling on the proportion of persons with true referable DR that attend follow-up at the central ophthalmology clinic
- The study benefits from a pragmatic design with broad inclusion criteria and involves the implementation of an AI system into a DR screening programme
- For practical reasons, it is not possible to mask participants to their arm allocation
- This study is not powered to investigate whether AI-supported screening increases treatment uptake or improves visual outcomes

For peer review only

INTRODUCTION

By 2030, non-communicable diseases (NCD) are set to overtake communicable, maternal, neonatal, and nutritional diseases combined as the leading cause of mortality in sub-Saharan Africa (SSA).[1] The rising number of people with diabetes is a major contributor to this and from 2021 to 2045 the number of people with diabetes is projected to rise by 129% in SSA.[2] This is proportionally more than any other region of the world.[2, 3]

Diabetic retinopathy (DR) is a common complication of diabetes and is a leading cause of blindness globally.[4] DR is asymptomatic in its early stages and often only begins to affect vision when the disease is at an advanced stage when is much less likely to respond to treatment; therefore screening is recommended to detect potentially sight-threatening DR and to refer people for assessment and treatment by an ophthalmologist. Early detection and timely treatment of sight threatening DR can prevent blindness in 95% of people with this stage of the disease.[5]

Several countries have successfully introduced DR screening programmes. For example, in the UK, following the introduction of a national DR screening programme, DR is no longer the leading cause of blindness in working-aged adults.[6] However, in many low- and middle-income countries (LMICs) there are no or very limited DR screening services and those that do exist suffer from several challenges.

At 12.3%, Tanzania is estimated to have the highest age-adjusted prevalence of diabetes in adults in Africa;[2] this has increased from an estimated prevalence of 2.3% in 2011.[2] In 2018 the Tanzanian Ministry of Health published guidelines which recommend annual screening of persons living with diabetes for DR.[7]

From 2010-2020 the Kilimanjaro Diabetic Programme (KDP) operated as a mobile community-based DR screening service across the Kilimanjaro region of Tanzania.[8] The screening team regularly visited each diabetic clinic and captured retinal photographs from people living with diabetes. The photographs were then graded at a later date (usually after a few weeks) by an ophthalmology resident (specialist doctor in training) at the Kilimanjaro Christian Medical Centre (KCMC) Eye Department (the referral hospital). Patients screened were then contacted with their results via text message or phone call and were given advice on whether they needed to attend KCMC.

Major challenges were faced by the KDP as well as other similar programmes in the African region. First, there are not enough skilled eye care staff. Often the same eye care staff screen, follow-up and then treat patients with sight-threatening disease, placing additional pressure on already over stretched systems. The number of eye care staff currently in the African region is already unable to cope with the clinical demands and is very likely to be overwhelmed by the projected surge in demand for diabetic eye care services over the next 30 years. Second, there is limited quality control and training of staff in grading retinal images, and it is therefore unclear how accurate the DR gradings are within the current screening programmes. And third, in the KDP, it was noted that there were poor rates of follow-up (42%) for those referred with potentially sight threatening DR from screening to secondary eye care.[8] This meant the KDP was identifying people with potentially sight-threatening DR at risk of sight loss, but less than half of those people attended the central ophthalmology clinic for assessment; this strongly affected the ultimate impact of the programme.

In Tanzania there is no widely available postal service and particularly older, poorer people have no or limited access to telephones and the internet, making it practically impossible to provide some patients with their results and follow-up advice at a later date. Moreover, through 300 interviews with diabetic patients registered with the KDP, we found that an understanding that DR can be

For peer review only - http://bmjopen.bmj.com/site/about/guidelines.xhtml

treated and knowledge of the location of the referral hospital are positively related to adherence to follow-up. The provision of an immediate referral decision with real-time feedback and point-of-screening counselling is likely to improve the rates of follow-up for those referred to the eye clinic with sight-threatening DR.[9, 10]

In view of the substantial projected increases in the number of people with diabetes worldwide, particularly in LMICs, there is an urgent need to test, develop and implement effective screening services for DR in order to prevent sight loss from the disease.

An alternative system to screen patients for DR that reduces demands on specialist staff, improves quality control and adherence to follow-up is needed. Advances in artificial intelligence (AI) for healthcare offer a potential alternative.

Ophthalmology is a leading medical specialty in healthcare AI, evidenced by the fact that the first autonomous medical AI device to get FDA approval (IDx-DR®) was for DR grading.[11] There are several AI systems commercially available for DR grading that have shown promisingly high sensitivities and specificities.[12] Such systems work by automating the interpretation of fundus photographs for DR, thereby reducing the need for trained clinical staff and, with high sensitivities and specificities, could enable the wide availability of high quality diagnostics within a short timeframe. Additionally, the immediate feedback provided by AI-supported screening software would enable a point-of-screening referral decision and counselling.

The current models of screening for DR do not work optimally in countries such as Tanzania. AI offers a promising solution to the specific problems faced in Tanzania, through decreased workforce pressures, improved quality control and potentially improved rates of follow-up for those people with sight threatening DR. However, in order for this technology to be appropriately and successfully implemented it needs to be tested and evaluated in prospective clinical trials.

AI-supported DR screening offers a potentially higher quality, lower unit cost and more accurate way to screen for DR, which is becoming an increasingly significant public health problem. To our knowledge, this study is the first independent trial in SSA to measure whether the implementation of an AI system into a DR screening programme with a point-of-screening result and face-to-face counselling can increase the proportion of persons with true referable DR that attend the central ophthalmology clinic. The results will contribute valuable evidence for considering the wider implementation of AI technologies for DR screening.

For peer review only

Objectives

The primary objective of this study is to determine if AI-supported DR screening can increase the proportion of people with true referable DR that attend the central ophthalmology clinic following referral after screening for assessment and treatment. The secondary objectives are: (1) to compare prospective programmatic sensitivity and specificity of AI-supported and standard DR screening for the detection of referable DR and (2) to determine the impact of AI-supported screening on other key screening programme indicators including false positive referrals, treatment uptake, gradable versus ungradable image capture and to undertake an economic evaluation.

For peer review only - http://bmjopen.bmj.com/site/about/guidelines.xhtml

METHODS AND ANALYSIS

Trial Summary

In an individually randomised controlled trial, we will test the hypothesis that AI-supported DR screening increases the proportion of people with true referable DR who attend the specialist eye clinic for assessment and treatment following referral after screening.

Persons with diabetes attending screening for DR will be randomised to either the current practice DR screening pathway or the AI-supported DR screening pathway (Figure 1). The AI-supported screening arm will include automated retinal image analysis with an immediate referral decision and point-of-screening counselling. We will assess the proportion of people with true referable DR who attend a follow-up appointment at the central ophthalmology clinic within eight weeks of screening.

Figure 1; Overview of the trial

AI, artificial intelligence; QALY, quality adjusted life year

Trial Setting

The trial will be conducted in the Kilimanjaro and Arusha regions of northern Tanzania. KCMC Eye department is a tertiary referral centre with a full range of ophthalmic specialists including medical retina, vitreo-retinal, glaucoma, paediatric, cornea and cataract specialists. As well as fundus cameras, the department has OCT imaging and both retinal laser and anti-VEGF treatments for DR are available. KCMC Eye department is where patients will be referred after screening if they have referable DR.

Eligibility Criteria

Potential participants will need to meet the eligibility criteria stipulated in Table 1. In summary, potentially eligible participants include all persons aged over 18 years with diabetes in the Kilimanjaro or Arusha regions of Tanzania and who attend a diabetic clinic, which is where DR screening occurs. The eligibility criteria are broad to reflect the reality of DR screening in Tanzania.

Table 1; Inclusion and exclusion criteria for enrolment into the randomised controlled trial

Inclusion criteria	Exclusions criteria (any of the following)
1. Adult (18 years and older) living with diabetes and attending diabetes clinics in Kilimanjaro or Arusha regions	1. Unable or unwilling to consent
2. Willing and able to give consent	2. Less than 18 years old
3. Agree to be randomised to either AI-supported or standard of care DR screening	3. Already attending the central ophthalmology clinic or had a diabetic eye exam in the previous 12 months

Informed consent

Any patients who may be eligible to participate will be given a participant information sheet and its contents will be read out to them by a study team member. They will be asked if they would be willing to participate and it will be explained to the potential participant that depending on which arm of the trial they are randomised to, they will either receive their result on the day of screening or some weeks later.

For peer review only - http://bmjopen.bmj.com/site/about/guidelines.xhtml

The patient will then have the opportunity to discuss any questions they might have. If the patient agrees to participate, they will be asked to read and sign or place a thumb print on the study consent form (see supplementary file 1).

Clinical assessment

Following assessment of eligibility criteria and the informed consent process, the participant will proceed with the clinical assessment (Table 2). In summary, it will include a basic demographic and clinical history, visual acuity testing, blood sugar and blood pressure measurement followed by fundus photography.

Table 2; Demographic and clinical data

Assessment	Details
Demographic history	Sex, age, education level, occupation, self-reported tribe / ethnicity, health insurance status
Clinical history	Type of diabetes, duration of diabetes, current diabetes treatment (diet, medications, insulin), smoking history, alcohol consumption, known hypertension, any known diabetes complications, previous heart attack or stroke,
Visual acuity	Presenting best corrected VA. VA will be measured using a Tumbling-E logMAR chart with the PEEK VA smart phone application[13] by a trained study team member, for each eye separately.
Blood pressure	BP will be measured using a calibrated A&D (A&D Instruments Ltd, Abingdon, UK) UA-651 BP monitor.[14] Using the WHO STEPwise Approach to NCD risk factor Surveillance protocol,[15] three measurements will be taken one minute apart after the participant has rested for five minutes, with an average of the final two measurements recorded.
Random blood sugar	A random blood sugar measurement will be taken using a Contour Next Meter (Ascenia Diabetes Care Limited, UK)[16] and recorded in mmol/L.
Fundus photography	One drop of tropicamide 1% will be instilled into each eye separately. After 30 minutes (to allow adequate pupil dilation) a Topcon NW8 fundus camera will be used to capture two retinal photographs of each eye: one disc centred image and one macular centred image. The retinal images will be captured by a trained medical photographer.

VA = visual acuity; BP, blood pressure; NCD, non-communicable disease

For peer review only

Intervention and standard of care

Standard of care

The standard of care is the process by which patients have been screened for DR in the Kilimanjaro DR screening programme for >10 years. After retinal image capture, the photographs will be stored on a laptop and taken back to KCMC eye department. The images will be graded by an ophthalmology resident for the level of DR. Persons with referable DR will be contacted by text message or phone call some weeks after screening and advised to attend KCMC eye department for assessment with or without treatment. The counselling participants in the standard of care arm receive will follow the same format as the counselling given in the intervention arm of the trial, except that it will be delivered by telephone.

Intervention arm

The intervention in this trial is a complex intervention[17] including AI-supported retinal image analysis for DR grading with immediate feedback and referral advice followed by point-of-screening patient counselling.

For peer review only - http://bmjopen.bmj.com/site/about/guidelines.xhtml

There are two components to the intervention:

- AI retinal image analysis and DR grading
- Face-to-face counselling at the point of screening

The use of AI to grade retinal images for DR enables point-of-screening, face-to-face counselling as automated analysis provides immediate feedback after image submission, with a referral decision. The screening team member will inform the participant of their DR grade and proceed with the counselling component of the intervention. The point-of-screening counselling will include an explanation of what DR is, the potential to cause vision loss, the referral process and will emphasise that there are effective and safe treatments for DR.

AI system selection

There are several AI systems commercially available for DR screening.[12] A review process informed the choice of AI system in this trial.[18] In summary the following criteria were considered: existing regulatory approval (FDA approval or CE marking), ability to work offline, evidence of performance in an African population and the ability to work with different cameras. The SELENA+ AI software (EyRIS Pte Ltd)[19] was the only system to meet all these criteria and was therefore selected. In a prior published validation study in Zambia the SELENA+ AI system had a sensitivity of 92.25% and a specificity of 89.04% for detecting referable DR.[20]

Counselling component of the intervention

In order to develop a clear, concise and contextually appropriate counselling script to be used to inform participants of their screening outcome, two focus group discussions (FGD) have been held. Participants in the FGDs included persons living with diabetes in the Kilimanjaro region and who are eligible to participate in the trial. A description of the research was provided and the participants were specifically asked what information they would like to be told when receiving their screening result. The counselling component of the intervention will be piloted with persons living with diabetes in the Kilimanjaro region prior to the start of the trial.

To ensure that the same information is shared in a similar form of words in both trial arms, the same counselling script will be used when patients are given their screening result irrespective of their allocation.

Randomisation

Randomisation sequence

A computer-generated randomisation list will be prepared by a statistician at London School of Hygiene & Tropical Medicine (LSHTM). The sequence will be in a 1:1 allocation ratio of standard DR screening to AI-supported DR screening, blocked with a random block size of 4 to 8.

Allocation concealment

The randomisation sequence will be concealed in sequentially numbered, opaque envelopes. The envelopes will be prepared by an administrator who is independent of all other aspects of the trial. A trained research team member will be responsible for opening the next envelope in numbered sequence and allocating the patient to either the AI-supported or standard of care DR screening pathway.

For peer review only - http://bmjopen.bmj.com/site/about/guidelines.xhtml

Masking

As immediate feedback is provided in the intervention arm versus no feedback at the point of screening in the standard of care arm, it will not be possible to mask participants or screening staff to the allocation. However, the arm the participant is allocated to will only be apparent after randomisation has occurred. The primary outcome is attendance at the central ophthalmology clinic within eight weeks of screening for those with true referrable DR; the research assistant collecting this information at KCMC eye clinic will be masked to the allocation.

Grading of retinal photographs

All retinal images captured in the trial will be graded by (1) the Tanzanian ophthalmology residents (2) the AI system and (3) UK certified graders. This will enable comparison of the performance of the AI system versus Tanzanian ophthalmology residents and against a reference standard (UK certified graders). All graders will be masked to the other DR gradings.

For the reference standard gradings, the UK certified graders will undertake feature based grading and the retinal images will be labelled using a software programme (OptoMize®, NEC Software Solutions, UK) commonly used in the English National Screening Programme. All images will be labelled by two experienced graders from the English National Screening Programme. The graders will only have access to anonymised retinal images with no patient metadata. Any disagreements will be adjudicated by a third senior grader. All graders involved are subject to the English Diabetic Eye Screening quality assurance process.

Participants noted to have any of the following after grading by the UK certified graders will be defined as having true referable DR: pre-proliferative, proliferative DR, referable maculopathy or an ungradable retinal image. Referable maculopathy is defined as the presence of hard exudates or blot haemorrhage within 1 disc diameter of the fovea (centre of the retina) or a circinate or group of exudates within the macula. This definition of referable DR and maculoapthy follows the Tanzanian National Guidelines for the Management of DR (published by the Tanzanian Ministry of Health) which is the nationally approved standard of care for Tanzania.

Follow-up assessment

All participants screened in the trial will be tracked to determine whether or not they attend the central ophthalmology clinic. Any participant recommended to attend a follow-up appointment who has not done so within eight weeks of screening will be considered a non-attender. A follow-up period of less than eight weeks could bias in favour of the intervention as some participants in the standard of care arm may not receive their screening result until three to four weeks after screening. As attendance at the central ophthalmology clinic beyond eight weeks becomes increasingly less attributable to the DR screening result, it is our view that attendance within eight weeks of screening is most appropriate.

For participants that attend the ophthalmology clinic we will document what, if any, treatment they are recommended and whether they commence the recommended treatment.

Lost to follow-up

As the primary outcome is attendance or not at the referral hospital within eight weeks of screening any persons not attending follow-up will be analysed as such.

For peer review only - http://bmjopen.bmj.com/site/about/guidelines.xhtml

Outcome measures

Primary outcome measure

The primary outcome will be the proportion of persons with true referable diabetic retinopathy who attend the central ophthalmology clinic within eight weeks of screening out of all those with true referable DR (defined by the reference standard), by trial arm.

All retinal images will be graded by UK certified graders to provide the reference standard. It will be these gradings that will determine which participants have true referable DR in each trial arm and this figure will be the denominator in our primary outcome analysis.

Secondary outcome measures

1. The proportion of persons that attend the central ophthalmology clinic within 8 weeks of screening out of all those referred, by trial arm
2. Sensitivity and specificity for grading referable DR
3. Number of false positive cases attending the central ophthalmology clinic, by trial arm
4. Number of patients receiving treatment after referral, by trial arm
5. Number of gradable versus ungradable retinal images
6. Time to presentation at hospital
7. Incremental cost per quality-adjusted life year (QALY) gained
8. Acceptability, appropriateness and fidelity of AI screening.

Data collection, management, confidentiality and access to data

Demographic and clinical data will be collected electronically in the field using a tablet (Huawei MediaPad T3; Model: KOB-WO9). The data collection forms will be developed using ODK software which is accessible via the tablet. ODK is a free open-source software fully encrypted for collecting, managing and using data is resource-constrained settings and works offline.[21] Data confidentiality will be maintained through restricted access to the data which will be password protected.

The retinal photographs collected in the study will be transferred onto an encrypted laptop after capture. Only authorized members of the study team will have access to these images. Any images transferred out of Tanzania for grading in the UK, in order to provide the reference standard, will be fully anonymised and labelled with internal linking IDs prior to transfer.

Data and safety monitoring board

The data and safety monitoring board (DSMB) for this trial comprises independent experts in statistics and ophthalmology appointed by the trial steering committee. The DSMB will meet bi-annually in person or virtually as needed to review the progress of the trial. Any modifications to the study protocol will be reviewed by the relevant ethics committees in the UK and Tanzania and the DSMB. The DSMB will also monitor any adverse events that occur during the trial.

Sample size considerations

This study is powered to test the hypothesis that AI-supported DR screening increases the proportion of persons with true referrable DR that attend the central ophthalmology clinic following referral after screening.

For peer review only

Our prior evaluation of the DR screening programme showed that the rate of follow-up for persons referred from screening to the ophthalmology clinic was 42%.[8] Using this value, and in discussion with our partners in Tanzania, it was agreed that a 33% increase in compliance with follow-up recommendations would be a significant and programmatically meaningful improvement to the service.

Assuming that 22.5% of persons screened have true referrable DR, with 1182 persons screened per trial arm (2364 persons in total), and therefore 266 persons with true referrable DR in each arm, we would have a 90% power to detect a 33% uplift (from 42% to 56%) in attendance at the ophthalmology clinic for those persons with true referable DR.

Analysis plan

The analysis will be by intention-to-treat (ITT) and all participant data will be analysed according to their randomisation allocation. However, not all participants randomised will be included in all analyses. For example, our primary outcome analysis includes only those persons with true referable DR as defined by the reference standard gradings.

The data will be analysed and reported according to the Consolidated Standards of Reporting Trials AI extension guidelines for reporting RCTs.[22] These guidelines are based on the CONSORT 2010 guidelines and have been adapted to produce a set of recommendations for clinical trials evaluating interventions with an AI component.

Primary outcome analysis: unadjusted analysis

The primary analysis will be a comparison of the proportion of persons with true referable DR out of all those with true referable DR (determined by the reference standard gradings) who attend the central ophthalmology clinic between the standard of care DR screening pathway (control) and the AI supported DR screening pathway (intervention). The primary analysis of the primary outcome will be by logistic regression model using attendance at eight weeks as the binary outcome, trial arm as the exposure and all participants who were classed as having referable DR by the reference standard measure will be included in the analysis. The intervention effect of AI-supported screening will be estimated as an odds ratio with a 95% confidence interval.

Primary outcome analysis: adjusted analysis

We will perform an adjusted analysis, adjusting for measured potential confounders including gender, education level and distance from the screening clinic to the referral hospital, to account for any potential imbalance between the arms in terms of measured variables (or associated unmeasured confounders).

As attendance versus non-attendance at the specialist eye clinic for participants with true referable DR is the primary outcome measure, missing outcome data due to loss to follow-up will not be an issue. For example, if an individual enrolled in the trial is referred to the central ophthalmology clinic but does not attend the follow-up appointment, they will be considered a non-attender and will be included in the analysis as such; they will not be considered lost to follow-up.

Analysis of other potential determinants for attendance of true referrable diabetic retinopathy cases at the eye clinic

For peer review only - http://bmjopen.bmj.com/site/about/guidelines.xhtml

For peer review only

Logistic regression models will be used to analyse potential factors that may be associated with the primary outcome (attendance of true referable DR cases at the eye clinic). These potential factors include: age, gender, education level, occupation, distance from screening site to referral hospital, type of diabetic clinic (government funded, private, church-based), duration of diabetes, visual acuity, type of diabetic treatment (insulin, tablets) and any previous ophthalmic treatments.

We will initially perform a univariable analysis of all variables, followed by a multivariable analysis adjusting for all potential confounders. Both the univariable and multivariable analyses will be presented.

Secondary outcome analysis

The secondary outcomes will be analysed as detailed in Table 3. Binary outcomes will be analysed using logistic regression and continuous variables with linear regression. The sensitivity and specificity for referable DR of the AI system and the Tanzanian ophthalmology residents will be reported by arm and for all participants.

Table 3; Secondary outcome measures and analyses

Secondary outcome measure	Analysis details
Proportion of persons that attend the central ophthalmology clinic within 8 weeks of screening out of all those referred	We will measure this outcome, by trial arm, in the same manner as our primary outcome measure. This will be by logistic regression with attendance at 8 weeks the binary outcome and trial arm the exposure. However, the denominator for this outcome measure will be all persons referred in each trial arm, in contrast to only persons with true referable DR in our primary outcome analysis.
Sensitivity and specificity for grading referable DR	All retinal images collected in the study will be graded by the AI system, the Tanzanian ophthalmology residents and the UK certified graders (reference standard). This will enable us to calculate the sensitivity and specificity of both the AI system and the local Tanzanian ophthalmologists for grading referable DR, relative to the reference standard.
False positive cases attending the central ophthalmology clinic	The number of false positive cases attending the central ophthalmology clinic will be compared between trial arms by logistic regression, with false positive cases attending the ophthalmology clinic as the outcome measure and trial arm the exposure.
Number of patients receiving treatment after referral	The study is not powered to detect a difference in treatment uptake but we will undertake an exploratory analysis using logistic regression to compare treatment uptake between the trial arms for those participants that attend the ophthalmology clinic and who are recommended treatment.
Number of gradable versus ungradable retinal images	The number of images deemed ungradable will be compared between trials arms by logistic regression with gradable / ungradable retinal image as the outcome and trial arm the exposure.
Time to presentation at hospital	The time to presentation at hospital will be compared between trial arms for all those referred to the central ophthalmology clinic after screening. We will use Cox proportional hazards regression with trial arm as the primary predictor. Survival curves using Kaplan-Meier will be plotted for both trial arms to represent the time to presentation at hospital.

For peer review only - http://bmjopen.bmj.com/site/about/guidelines.xhtml
For peer review only

Incremental cost per QALY gained	The economic evaluation aims to assess the cost effectiveness of an AI supported screening model compared to standard of care model. Cost-effectiveness results will be expressed in terms of incremental cost-effectiveness ratios (ICERs) per QALY gained.<br><br>A Markov model will be built to undertake the analysis. Both deterministic and probabilistic sensitivity analyses will be performed to test the robustness of the model. Uncertainty in the parameters in the model will be reflected using probability distributions and the overall decision uncertainty will be presented using cost-effectiveness acceptability curves.
Acceptability, appropriateness and fidelity of AI screening	A process evaluation will be embedded within the trial and will be undertaken using the Medical Research Council guidance.[23] Using semi-structured interviews with participants, screening team staff, ophthalmologists and Ministry of Health officials we will assess the acceptability of AI-supported DR screening from different perspectives (patients, staff, policy makers). These qualitative data will be analysed using Nvivo according to a condensation of meaning analysis and units of meaning will be coded into central themes.<br><br>The fidelity of AI-supported screening will be assessed during the trial by random spot checks evaluating the delivery of the intervention. Staff members will be asked to keep a log of any difficulties encountered whilst delivering the intervention, thereby highlighting implementation barriers and challenges.

DR, diabetic retinopathy. QALY, quality-adjusted life year.

Interim analysis

As there is negligible risk to participants an interim analysis is not required and data analysis will take place at the end of the study.

All retinal images collected during the study will be graded by local Tanzanian ophthalmology residents. Any images that are graded as referrable DR by the Tanzanian graders but were graded as non-referable by the AI (potential false negatives) will be reviewed by a senior grader. If the senior grader concludes that the participant does meet the referral threshold, participants will be contacted and advised to attend the eye clinic, thereby ensuring minimal risk to patients.

In such circumstances, the initial result from the AI grading will be used in the analysis and would therefore not affect the results.

Table 4; Trial Registration summary

Data Category	Information
Primary registry and trial indentifying no	ISRCTN Registry; ISRCTN18317152
Date of registration in primary registry	March 2nd 2023
Secondary identifying numbers	
Sources of monetary support	British Council for the Prevention of Blindness, CBM and the Sir Halley Stewart Trust
Primary sponsor	London School of Hygiene & Tropical Medicine
Secondary sponsor	

For peer review only - http://bmjopen.bmj.com/site/about/guidelines.xhtml

Contact for queries	Dr Charles Cleland; charles.cleland@lshtm.ac.uk
Title	Artificial intelligence support diabetic retinopathy screening in Tanzania
Countries of recruitment	Tanzania
Health condition(s) or problem(s) studies	Diabetic retinopathy
Intervention(s)	Artificial intelligence-supported retinal image analysis for diabetic retinopathy grading with immediate feedback and referral advice followed by point-of-screening patient counselling.
Key eligibility criteria	1. Adult (18 years and older) living with diabetes and attending a diabetic clinic<br>2. Agree to be randomised to either the AI-supported diabetic screening pathway or the standard of care diabetic retinopathy screening pathway
Study type	Randomised controlled trial
Date of first enrolment	March 8th 2023
Target sample size	2364
Recruitment status	Recruiting
Primary outcome(s)	Proportion of persons with true referable diabetic retinopathy who attend the central ophthalmology clinic within eight weeks of screening out of all those with true referable diabetic retinopathy
Key secondary outcomes	1. The proportion of persons that attend the central ophthalmology clinic within 8 weeks of screening out of all those referred, by trial arm<br>2. Sensitivity and specificity for grading referable DR<br>3. Number of false positive cases attending the central ophthalmology clinic, by trial arm<br>4. Number of patients receiving treatment after referral, by trial arm<br>5. Number of gradable versus ungradable retinal images<br>6. Time to presentation at hospital<br>7. Incremental cost per quality-adjusted life year (QALY) gained

For peer review only

Patient and public involvement

Our previous research highlighted the importance of knowledge of the referral process and the fact that DR can be treated as key factors that impact referral adherence,[24] both of which can be stated during point-of-screening counselling. We have held two focus group discussions with persons living with diabetes in the Kilimanjaro region, and who would be eligible for DR screening; the participants expressed a preference for a point-of-screening result over a delayed result.

Ethics and dissemination

Full ethical approval has been received from the London School of Hygiene & Tropical Medicine (LSHTM), UK and the Kilimanjaro Christian Medical Centre and the National Institute for Medical Research ethics committees in Tanzania. Any protocol modifications will be submitted to the relevant parties for review and / or approval. At the end of the study period any patients who still require treatment or follow-up will continue to be treated at KCMC Hospital. The trial has been registered with the ISRCTN clinical trials registry and the LSHTM is the trial sponsor (Table 4). Data will be available upon request from the corresponding author on completion of the trial.The results of this trial will be submitted for publication in peer reviewed journals and will be presented at local and international meetings.

For peer review only - http://bmjopen.bmj.com/site/about/guidelines.xhtml

References

1. *World Health Organization Regional Office of Africa. Non-communicable diseases. 2014. Available at:* *https://www.afro.who.int/health-topics/noncommunicable-diseases*.
2. Federation, I.D., *IDF Diabetes Atlas, 10th edn. Brussels, Belgium.* 2021.
3. Atun, R., et al., *Diabetes in sub-Saharan Africa: from clinical care to health policy.* Lancet Diabetes Endocrinol, 2017. **5**(8): p. 622-667.
4. Burton, M.J., et al., *The Lancet Global Health Commission on Global Eye Health: vision beyond 2020.* Lancet Glob Health, 2021. **9**(4): p. e489-e551.
5. Vujosevic, S., et al., *Screening for diabetic retinopathy: new perspectives and challenges.* Lancet Diabetes Endocrinol, 2020. **8**(4): p. 337-347.
6. Scanlon, P.H., *The English national screening programme for sight-threatening diabetic retinopathy.* J Med Screen, 2008. **15**(1): p. 1-4.
7. Bashir R, S.F., Peto T, Shilio B, Zondervan M, *The Diabetic Eye Screening Programme in Tanzania — The VISION 2020 LINK between Dodoma and Belfast.* Eye News, 2021. **27**.
8. Cleland, C.R., et al., *Diabetic retinopathy in Tanzania: prevalence and risk factors at entry into a regional screening programme.* Trop Med Int Health, 2016. **21**(3): p. 417-26.
9. Liu, J., et al., *Diabetic Retinopathy Screening with Automated Retinal Image Analysis in a Primary Care Setting Improves Adherence to Ophthalmic Care.* Ophthalmol Retina, 2021. **5**(1): p. 71-77.
10. Mathenge, W., et al., *Impact of Artificial Intelligence Assessment of Diabetic Retinopathy on Referral Service Uptake in a Low-Resource Setting: The RAIDERS Randomized Trial.* Ophthalmology Science, 2022. **2**(4): p. 100168.
11. Abramoff, M.D., et al., *Pivotal trial of an autonomous AI-based diagnostic system for detection of diabetic retinopathy in primary care offices.* NPJ Digit Med, 2018. **1**: p. 39.
12. Grzybowski, A., et al., *Artificial intelligence for diabetic retinopathy screening: a review.* Eye, 2020. **34**(3): p. 451-460.
13. Bastawrous, A., et al., *Development and Validation of a Smartphone-Based Visual Acuity Test (Peek Acuity) for Clinical Practice and Community-Based Fieldwork.* JAMA Ophthalmology, 2015. **133**(8): p. 930-937.
14. Benetti, E., C. Fania, and P. Palatini, *Validation of the A&D BP UA-651 device for home blood pressure measurement according to the European Society of Hypertension International Protocol revision 2010.* Blood Press Monit, 2014. **19**(1): p. 50-3.
15. *World Health Organization. STEPwise approach to Noncommunicable Disease Surveillance (STEPS).* 2017.
16. Harrison, B., C. Leazenby, and S. Halldorsdottir, *Accuracy of the CONTOUR® blood glucose monitoring system.* J Diabetes Sci Technol, 2011. **5**(4): p. 1009-13.
17. Skivington, K., et al., *Framework for the development and evaluation of complex interventions: gap analysis, workshop and consultation-informed update.* Health Technol Assess, 2021. **25**(57): p. 1-132.
18. Cleland, C.R., et al., *Artificial intelligence for diabetic retinopathy in low-income and middle-income countries: a scoping review.* BMJ Open Diabetes Res Care, 2023. **11**(4).
19. *EyRIS Pte Ltd*. Available from: https://www.eyris.io.
20. Bellemo, V., et al., *Artificial intelligence using deep learning to screen for referable and vision-threatening diabetic retinopathy in Africa: a clinical validation study.* Lancet Digit Health, 2019. **1**(1): p. e35-e44.

For peer review only - http://bmjopen.bmj.com/site/about/guidelines.xhtml

21. *Open Data Kit*. Available from: https://getodk.org.
22. Liu, X., et al., *Reporting guidelines for clinical trial reports for interventions involving artificial intelligence: the CONSORT-AI extension.* Nature Medicine, 2020. **26**(9): p. 1364-1374.
23. Moore, G.F., et al., *Process evaluation of complex interventions: Medical Research Council guidance.* Bmj, 2015. **350**: p. h1258.
24. Mtuya, C., et al., *Reasons for poor follow-up of diabetic retinopathy patients after screening in Tanzania: a cross-sectional study.* BMC Ophthalmol, 2016. **16**: p. 115.

For peer review only

For peer review only - http://bmjopen.bmj.com/site/about/guidelines.xhtml

Author Statement: Searched the literature: CRC. Drafted initial protocol: CRC. Contributed to protocol development and revision: CRC, CB, WM, BS, FS, HP, APM, CE, AT, AD, PK, DM, MJB. Drafted this manuscript: CRC. Critically revised this manuscript: CRC, CB, WM, BS, FS, HP, APM, CE, AT, AD, PK, DM, MJB. Conceptualisation: CRC. Funding acquisition: CRC.

Funding: This research was supported by the British Council for the Prevention of Blindness, Christian Blind Mission and the Sir Halley Stewart Trust. PK is supported by a Moorfields Eye Charity Career Development Award (R190028A) and a UK Research & Innovation Future Leaders Fellowship (MR/T019050/1). MJB is supported by the Wellcome Trust (207472/Z/17/Z).

Disclaimer: The views expressed are those of the authors and not necessarily those of the NIHR or the Department of Health and Social Care. The funders have had, and will have, no role in the study design, data collection and analysis, decision to publish or preparation of the macuscript.

Competing interests: CE has received fees from Heidelberg Engineering, Inozyme Pharma. AT has received fees from Annexon, Apellis, Bayer, Genentech, Iveric Bio, Novartis, Oxurion, and Roche.PK has acted as a consultant for Roche, Novartis, Boehringer-Ingleheim, Adecco, Bitfount and is an equity owner in Big Picture Medical. PK has received speaker fees from Novartis, Gyroscope, Bayer, Thea, Boehringer-Ingleheim, Apellis, Abbvie, Alimera, Roche, Genentech, Specsavers, Heidelberg Engineering, Topcon, and Santen. PK has received travel support from Bayer, Topcon, and Roche. PK has attended advisory boards for Boehringer-Ingleheim, RetinAI, Novartis, Apellis, Abbvie, and Roche. No other authors declare any conflicts.

Trial sponsor: London School of Hygiene & Tropical Medcine, Keppel Street, London. WC1E 7HT

Acknowledgments: The authors would like to thank the Kilimanjaro Christian Medical Centre Eye Department for their support with study co-ordination and implementation. The authors would like to thank the EyRIS PTE Ltd team for their support with providing the AI software, in particular Steven Ang. The authors would like to thank the staff at the Eye Screening Centre at the Homerton Hospital, in particular John Anderson and Louis Bolter, for their support with providing the reference standard gradings.

For peer review only

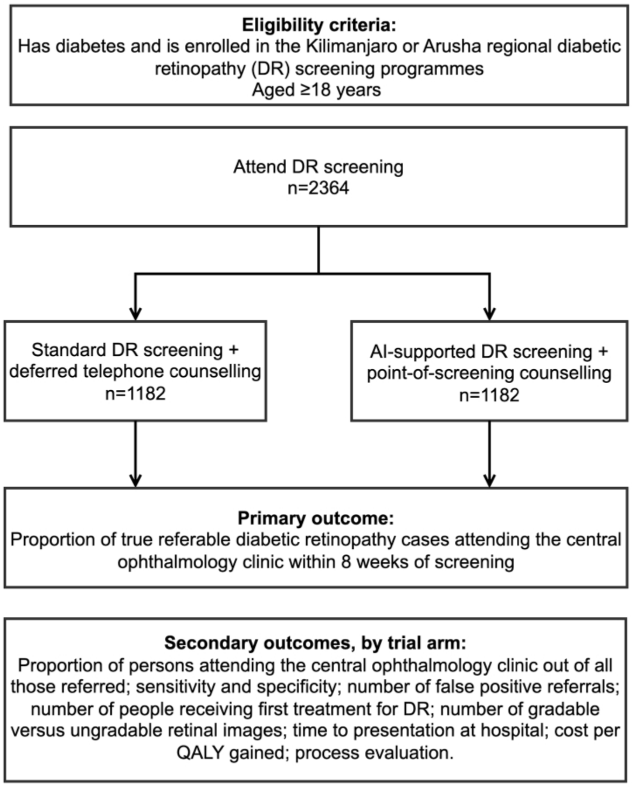

AI, artificial intelligence; QALY, quality adjusted life year

110x156mm (800 x 800 DPI)

Adult Consent Form

Study Title:
Artificial Intelligence Supported Diabetic Retinopathy Screening in Tanzania

- I confirm that I have read the information sheet dated 30/5/2022 (version 1.1) for the above study. I have had the opportunity to consider the information, ask questions and have had these answered satisfactorily. ☐
- I understand that my participation is voluntary and that I am free to withdraw at any time without giving any reason, without my medical care or legal rights being affected. ☐
- I understand that the information/retinal photographs collected about me may be used to support other research in the future, and may be shared anonymously with other researchers. ☐
- I agree to receive individual feedback about my condition ☐
- I understand that the information held and maintained by the Kilimanjaro Christian Medical Centre eye department may be used to help contact me or provide information about my health status. ☐
- I agree to take part in the above study. ☐

Participant:

Signature: ……………………………………………………………………………………………

Print name: …………………………………………………………………………………………..

Date:……………………………………………………………………………………………

AI-supported DR screening in Tanzania – main trial consent form – V1.1 – 30/05/2022

National Health Research Ethics Review Committee
National Institute for Medical Research
2448 Ocean Road, P.O. Box 9653
Dar es Salaam, Tanzania
Tel: +255 22 2121400
Website: www.nimr.or.tz

For peer review only

Witness:

Signature: …………………………………………………………………………………

Print name: ………………………………………………………………………………..

Date:…………………………………………………………………………………………

Second witness (if required):

Signature: …………………………………………………………………………………

Print name: ………………………………………………………………………………..

Date:…………………………………………………………………………………………

For peer review only

AI-supported DR screening in Tanzania – main trial consent form – V1.1 – 30/05/2022

National Health Research Ethics Review Committee
National Institute for Medical Research
2448 Ocean Road, P.O. Box 9653
Dar es Salaam, Tanzania
Tel: +255 22 2121400
Website: www.nimr.or.tz

SPIRIT-AI

SPIRIT-AI Checklist: Recommended items to address in a protocol and related documents for clinical trials evaluating AI interventions

Section	SPIRIT 2013 Item[a]		SPIRIT-AI Item		Addressed on Page No [b]
**Administrative Information**					
Title	1	Descriptive title identifying the study design, population, interventions, and, if applicable, trial acronym	SPIRIT-AI 1(i) Elaboration	Indicate that the intervention involves artificial intelligence / machine learning and specify the type of model.	1
			SPIRIT-AI 1(ii) Elaboration	Specify the intended use of the AI intervention.	1
Trial registration	2a	Trial identifier and registry name. If not yet registered, name of intended registry			2
	2b	All items from the World Health Organization Trial Registration Data Set			14
Protocol version	3	Date and version identifier			Not relevant for protocol submitted for publication
Funding	4	Sources and types of financial, material, and other support			17
Roles and responsibilities	5a	Names, affiliations, and roles of protocol contributors			1 and 17
	5b	Name and contact information for the trial sponsor			17
	5c	Role of study sponsor and funders, if any, in study design; collection, management, analysis, and interpretation of data; writing of the report; and the decision to submit the report for publication, including whether they will have ultimate authority over any of these activities			17
	5d	Composition, roles, and responsibilities of the coordinating centre, steering committee, endpoint adjudication committee, data management team, and other individuals or groups overseeing the trial, if applicable (see Item 21a for data monitoring committee)			11
**Introduction**					
Background and rationale	6a	Description of research question and justification for undertaking the trial, including summary of relevant studies (published and unpublished) examining benefits and harms for each intervention	SPIRIT-AI 6a (i) Extension	Explain the intended use of the AI intervention in the context of the clinical pathway, including its purpose and its intended users (e.g. healthcare professionals, patients, public).	4-5 and 8-9

Cite as:

For peer review only - http://bmjopen.bmj.com/site/about/guidelines.xhtml

			SPIRIT-AI 6a (ii) Extension	Describe any pre-existing evidence for the AI intervention.	5
	6b	Explanation for choice of comparators			8
Objectives	7	Specific objectives or hypotheses			5
Trial design	8	Description of trial design including type of trial (eg, parallel group, crossover, factorial, single group), allocation ratio, and framework (eg, superiority, equivalence, noninferiority, exploratory)			6
Methods: Participants, Interventions and Outcomes					
Study setting	9	Description of study settings (eg, community clinic, academic hospital) and list of countries where data will be collected. Reference to where list of study sites can be obtained	SPIRIT-AI 9 Extension	Describe the onsite and offsite requirements needed to integrate the AI intervention into the trial setting.	7-8
Eligibility criteria	10	Inclusion and exclusion criteria for participants. If applicable, eligibility criteria for study centres and individuals who will perform the interventions (eg, surgeons, psychotherapists)	SPIRIT-AI 10 (i) Elaboration	State the inclusion and exclusion criteria at the level of participants.	7
			SPIRIT-AI 10 (ii) Extension	State the inclusion and exclusion criteria at the level of the input data.	7
Interventions	11a	Interventions for each group with sufficient detail to allow replication, including how and when they will be administered	SPIRIT-AI 11a (i) Extension	State which version of the AI algorithm will be used.	N/A – a commercially available AI system will be used.
			SPIRIT-AI 11a (ii) Extension	Specify the procedure for acquiring and selecting the input data for the AI intervention.	8
			SPIRIT-AI 11a (iii) Extension	Specify the procedure for assessing and handling poor quality or unavailable input data.	10
			SPIRIT-AI 11a (iv) Extension	Specify whether there is human-AI interaction in the handling of the input data, and what level of expertise is required for users.	8-9
			SPIRIT-AI 11a (v) Extension	Specify the output of the AI intervention.	8-9
			SPIRIT-AI 11a (vi) Extension	Explain the procedure for how the AI intervention's output will contribute to decision-making or other elements of clinical practice.	8-9
	11b	Criteria for discontinuing or modifying allocated interventions for a given trial participant (eg, drug dose change in response to harms, participant request, or improving/worsening disease)			13-14
	11c	Strategies to improve adherence to intervention protocols, and any procedures for monitoring adherence (eg, drug tablet return, laboratory tests)			8-9

Cite as:

SPIRIT-AI

	11d	Relevant concomitant care and interventions that are permitted or prohibited during the trial			N/A – not relevant to this study
Outcomes	12	Primary, secondary, and other outcomes, including the specific measurement variable (eg, systolic blood pressure), analysis metric (eg, change from baseline, final value, time to event), method of aggregation (eg, median, proportion), and time point for each outcome. Explanation of the clinical relevance of chosen efficacy and harm outcomes is strongly recommended			10
Participant timeline	13	Time schedule of enrolment, interventions (including any run-ins and washouts), assessments, and visits for participants. A schematic diagram is highly recommended (see Figure)			6-7
Sample size	14	Estimated number of participants needed to achieve study objectives and how it was determined, including clinical and statistical assumptions supporting any sample size calculations			11
Recruitment	15	Strategies for achieving adequate participant enrolment to reach target sample size			11
Methods: Assignment of Interventions (For Controlled Trials)					
Sequence generation	16A	Method of generating the allocation sequence (eg, computer-generated random numbers), and list of any factors for stratification. To reduce predictability of a random sequence, details of any planned restriction (eg, blocking) should be provided in a separate document that is unavailable to those who enrol participants or assign interventions			9
Allocation concealment mechanism	16b	Mechanism of implementing the allocation sequence (eg, central telephone; sequentially numbered, opaque, sealed envelopes), describing any steps to conceal the sequence until interventions are assigned			9
Implementation	16c	Who will generate the allocation sequence, who will enrol participants, and who will assign participants to interventions			9
Blinding (masking)	17a	Who will be blinded after assignment to interventions (eg, trial participants, care providers, outcome assessors, data analysts), and how			9
	17b	If blinded, circumstances under which unblinding is permissible, and procedure for revealing a participant's allocated intervention during the trial			N/A – single blinded study
Methods: Data Collection, Management, And Analysis					
Data collection methods	18a	Plans for assessment and collection of outcome, baseline, and other trial data, including any related processes to promote data quality (eg, duplicate measurements, training of assessors) and a description of study instruments (eg, questionnaires, laboratory tests) along with their reliability and validity, if known. Reference to where data collection forms can be found, if not in the protocol			10-11

Cite as:

For peer review only

	18b	Plans to promote participant retention and complete follow-up, including list of any outcome data to be collected for participants who discontinue or deviate from intervention protocols			10
Data management	19	Plans for data entry, coding, security, and storage, including any related processes to promote data quality (eg, double data entry; range checks for data values). Reference to where details of data management procedures can be found, if not in the protocol			10-11
Statistical methods	20a	Statistical methods for analysing primary and secondary outcomes. Reference to where other details of the statistical analysis plan can be found, if not in the protocol			11-13
	20b	Methods for any additional analyses (eg, subgroup and adjusted analyses)			11-13
	20c	Definition of analysis population relating to protocol non-adherence (eg, as randomised analysis), and any statistical methods to handle missing data (eg, multiple imputation)			11-13
Methods: Monitoring					
Data monitoring	21a	Composition of data monitoring committee (DMC); summary of its role and reporting structure; statement of whether it is independent from the sponsor and competing interests; and reference to where further details about its charter can be found, if not in the protocol. Alternatively, an explanation of why a DMC is not needed			11
	21b	Description of any interim analyses and stopping guidelines, including who will have access to these interim results and make the final decision to terminate the trial			13-14
Harms	22	Plans for collecting, assessing, reporting, and managing solicited and spontaneously reported adverse events and other unintended effects of trial interventions or trial conduct	SPIRIT-AI 22 Extension	Specify any plans to identify and analyse performance errors. If there are no plans for this, explain why not.	13-14
Auditing	23	Frequency and procedures for auditing trial conduct, if any, and whether the process will be independent from investigators and the sponsor			11
Ethics and Dissemination					
Research ethics approval	24	Plans for seeking research ethics committee/institutional review board (REC/IRB) approval			15
Protocol amendments	25	Plans for communicating important protocol modifications (eg, changes to eligibility criteria, outcomes, analyses) to relevant parties (eg, investigators, REC/IRBs, trial participants, trial registries, journals, regulators)			15
Consent or ascent	26a	Who will obtain informed consent or assent from potential trial participants or authorised surrogates, and how (see Item 32)			7

Cite as:

	26b	Additional consent provisions for collection and use of participant data and biological specimens in ancillary studies, if applicable			7
Confidentiality	27	How personal information about potential and enrolled participants will be collected, shared, and maintained in order to protect confidentiality before, during, and after the trial			10-11
Declaration of interests	28	Financial and other competing interests for principal investigators for the overall trial and each study site			17
Access to data	29	Statement of who will have access to the final trial dataset, and disclosure of contractual agreements that limit such access for investigators	SPIRIT-AI 29 Extension	State whether and how the AI intervention and/or its code can be accessed, including any restrictions to access or re-use.	N/A – a commercially available AI system will be used
Ancillary and post-trial care	30	Provisions, if any, for ancillary and post-trial care, and for compensation to those who suffer harm from trial participation			15
Dissemination policy	31a	Plans for investigators and sponsor to communicate trial results to participants, healthcare professionals, the public, and other relevant groups (eg, via publication, reporting in results databases, or other data sharing arrangements), including any publication restrictions			15
	31b	Authorship eligibility guidelines and any intended use of professional writers			17
	31c	Plans, if any, for granting public access to the full protocol, participant-level dataset, and statistical code			15
Appendices					
Informed consent materials	32	Model consent form and other related documentation given to participants and authorised surrogates			Appendix 1
Biological specimens	33	Plans for collection, laboratory evaluation, and storage of biological specimens for genetic or molecular analysis in the current trial and for future use in ancillary studies, if applicable			N/A – no biological specimens will be collected

[a] It is strongly recommended that this checklist be read in conjunction with the SPIRIT 2013 Explanation & Elaboration for important clarification on the items.
[b] Indicates page numbers to be completed by authors during protocol development.

Cite as:

For peer review only
